# Shift work, genetic risk, and incident gout: a prospective cohort study in the UK Biobank

**DOI:** 10.1186/s13690-026-01888-1

**Published:** 2026-03-17

**Authors:** Chuanghai Wu, Haiyun Zhang, Jinjin Xia, Baizhao Peng, Jieyu Chen, Ming Wang, Siqi Wu, Zihao Jiang, Shuai Ji, Ying Yang, Yanting You, Hiu Yee Kwan, Zhuhua Sun, Xiaoshan Zhao, Yanyan Liu

**Affiliations:** 1 Department of Traditional Chinese Medicine, Zhujiang Hospital, Southern Medical University, 253 Gongye Middle Avenue, Haizhu District, Guangzhou, Guangdong 510280 China; 2 School of Traditional Chinese Medicine, Southern Medical University, No.1023 South Shatai Road, Baiyun District, Guangzhou, Guangdong 510515 China; 3 National Institute of TCM Constitution and Preventive Medicine, Beijing University of Chinese Medicine, Beijing, 100029 China; 4 School of Chinese Medicine, Hong Kong Baptist University, Hong Kong, 999077 China; 5 The Third Affiliated Hospital of Southern Medical University, Guangzhou, Guangdong 510630 China

**Keywords:** Occupational health, Genetic predisposition, Gout, Hyperuricemia, Prospective cohort study

## Abstract

**Background:**

Shift work (SW) has been linked to the occurrence of various chronic diseases. However, its potential correlation with gout has not been established. This study aimed to investigate the effects of the SW schedule on incident gout.

**Methods:**

A total of 281,500 individuals enrolled in the UK Biobank were included in the cohort study. The Cox proportional hazards model was used to investigate the association between SW and incident gout. Additionally, a genetic risk score (GRS) was constructed to assess the joint effects of SW and genetic predisposition on the risk of gout.

**Results:**

During a 13-year follow-up period, we recorded 4,282 new-onset gout cases. In the current SW analysis, individuals with some night shifts had a 15.1% higher gout risk compared to day workers, which varied by smoking status, chronotype, and obesity status [hazard ratio (HR): 1.151, 95% confidence interval (CI): 1.018–1.301]. The lifetime analysis indicated that individuals with < 5 years of night shifts were more prone to gout than those without night shift exposure, especially when excluding those who developed gout within one year from the baseline (HR: 1.259, 95% CI: 1.027–1.542). An interaction between SW and genetic predisposition was observed, with individuals having both high GRS and some night shifts showing a 218% higher gout risk compared to those with low GRS and day work (HR: 3.18, 95% CI: 2.58–3.92).

**Conclusions:**

Both current and lifetime SW were associated with an increased gout risk, with genetic predisposition further amplifying this risk. These findings contribute additional evidence to the current understanding of the detrimental effects of SW and provide a novel and important perspective on strategies for the primary prevention of incident gout.

**Graphical Abstract:**

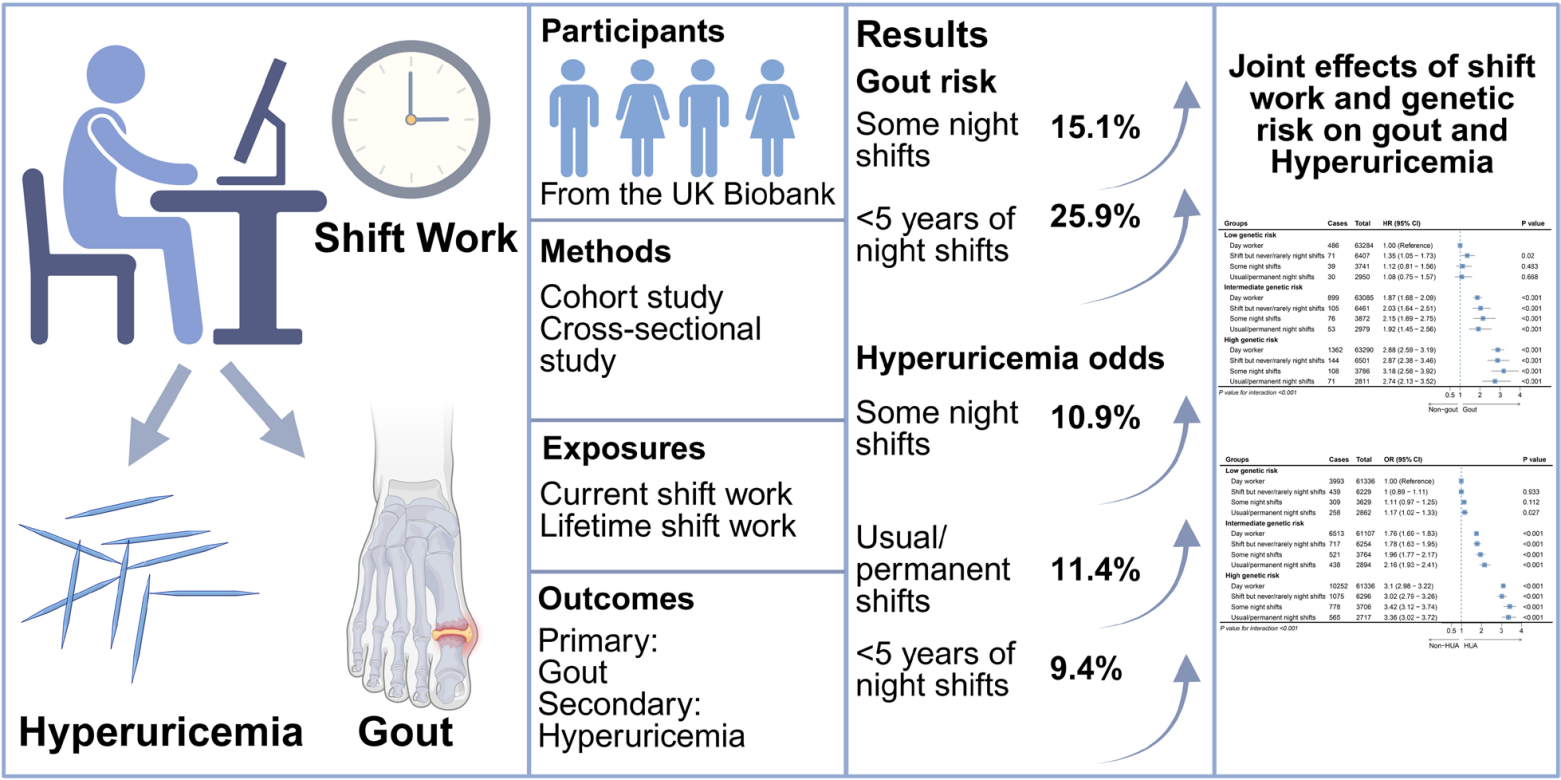

**Supplementary Information:**

The online version contains supplementary material available at 10.1186/s13690-026-01888-1.

**Table:** 

Text box 1. Contributions to the literature
• Shift work (SW) is increasingly common and linked to chronic health issues, but its impact on gout risk remains underexplored.
• This study identifies an association between SW and increased gout risk, with genetic factors further exacerbating this risk.
• These findings emphasize the need to consider SW as a modifiable factor in the prevention of gout and other chronic conditions, offering valuable insights for public health strategies.

## Introduction

Serum uric acid (SUA) is the end product of purine metabolism within the human body, and its elevated concentrations may lead to the development of hyperuricemia (HUA) and gout. Gout is a common chronic condition caused by the deposition of monosodium urate crystals and is characterized by intermittent painful arthritis [1]. Both the incidence and prevalence of gout showed an upward trend [2]. Except for typical clinical presentation (gout flare, tophi, chronic gout arthritis, etc.), gout is commonly associated with a higher prevalence of comorbidities, such as hypertension, cardiovascular disease, stroke, chronic kidney disease (CKD), etc [1, 3]. As indicated in the recommendations from the European League Against Rheumatism, the identification of risk factors is crucial for the management of gout patients [4]. Many factors contribute to the occurrence of gout, such as obesity [5], diuretics, and dietary factors [1]. Given the substantial and increasing population diagnosed with gout, the identification of the hidden risk factors is urgently needed.

In contemporary society, shift work (SW) has become a prevalent work schedule. In the U.S., 28.7% of the workers follow alternative shift schedules [6], which are typically associated with circadian disruption [7]. The disturbed circadian rhythm is the underlying mechanism that SW leads to the occurrence of multiple chronic diseases [8], such as diabetes [9], hypertension [10], and atrial fibrillation [11]. The presence of a circadian rhythm of SUA concentrations has also been observed within the healthy human body as well as in patients diagnosed with gout [12–14]. For example, the risk of experiencing an acute gout attack was 2.26 times higher at night compared to daytime [15]. It is hypothesized that the disrupted circadian rhythm contributes to the development of SUA-related conditions, including HUA and gout. However, whether SW, a key example of circadian disruption, is associated with increased susceptibility to gout and HUA in large populations remains unknown.

In this study, we first conducted a large community-based cohort study that included 281,500 participants based on the UK Biobank. We aimed to investigate the correlation between current and lifetime SW and the occurrence of gout, with gout being the primary outcome of interest. We also considered HUA as a secondary outcome and conducted a cross-sectional analysis to explore the potential association between current and lifetime SW and the prevalence of HUA. Secondly, we further investigated the joint effects of SW and genetic risk for SUA on the likelihood of incident gout and prevalent HUA.

## Methods

### Study design and population

UK Biobank is a large prospective cohort that recruited more than 500,000 participants aged 37–73 years across the United Kingdom from 2006 to 2010 (https://www.ukbiobank.ac.uk/). Details about the cohort have been reported in previous articles [16]. In the current analysis, we included those who were in paid employment or self-employed at baseline (*N* = 286,356). We excluded participants with prevalent gout at baseline (*N* = 4,856), and 281,500 were selected for cohort analysis. Among them, 229,176 individuals had available genetic data. We further selected 74,913 individuals (61,740 of them had genetic data) who had lifetime employment data for lifetime SW analysis (Fig. S1). In addition, we also explored possible associations between SW and HUA, and the inclusion and exclusion processes were summarized in Fig. S1. The methods and results in this study are reported following the Strengthening the Reporting of Observational Studies in Epidemiology (STROBE) statement.

### Exposures

In the UK Biobank, SW is defined as a work schedule involving “working afternoons, evenings or nights or rotating through these kinds of shifts”, which is different from the daytime work schedule (9 am to 5 pm). During the baseline period, participants who were currently employed were asked about their employment status and whether their current job involved a SW schedule. If so, participants were further asked whether their main job involved the night shift. In the UK Biobank, night shifts are defined as a work schedule in which participants work through the normal sleep period (12 am to 6 am). We divided the participants into four groups of SW based on the answers to the two questions, including day workers, shift but never/rarely night shifts, some night shifts, and usual/permanent night shifts [11].

As suggested previously [9], we calculated lifetime SW duration (i.e., the number of years working night shifts) and frequency (i.e., the number of average night shifts each month). The lifetime SW duration was categorized as none (reference group), < 5 years, 5–10 years, and > 10 years while the frequency was divided into none (reference group), < 3 nights/month, 3–8 nights/month, and > 8 nights/month.

### Outcomes

For the primary outcome, incident gout (filed ID 131859), was determined based on “first occurrence” (category ID 1712), incorporating records from primary care, hospital inpatient admissions, death registers, and self-reported medical conditions, as per previous studies [17–19]. For the secondary outcome, prevalent HUA was defined as serum urate (field ID 30880) higher than 420 µmol/L among men or 360 µmol/L among women [20].

### Weighted genetic risk score for serum uric acid

Details of the genotyping in the UK Biobank have been described in a previous study [21]. We constructed a weighted genetic risk score (GRS) based on 114 independent serum urate-related single nucleotide polymorphisms (SNPs) and their effects presented in a previous paper [22], which were provided in detail in Table S1. For each SNP, we calculated the number of urate-increasing alleles, which was weighted by the SNP effect size, and added the weighted score for all 114 SNPs [23]. Finally, the weighted GRS for the participants with available genetic data was generated, and they were divided into low-, intermediate-, and high-GRS groups.

### Covariates

The covariates we considered were divided into three parts. Part Ⅰ was demographic and socioeconomic information, including sex (male/female), age (continuous variable), household income [low (< £18,000), medium (£18,000-£51,999), and high (≥ £52,000)] [11], and education qualifications (college or above, high school or equivalent, and less than high school). Part Ⅱ was lifestyle factors, which contained smoking status (never, previous, and current), drinking frequencies (frequent, infrequent, and never) [24], and diet quality scores (0/1/2/3/4), calculated as per a previous study (details in the Supplementary information section) [25]. Diet quality was further categorized as poor (0–1), intermediate (2), and healthy (3–4). Part Ⅲ was composed of several chronic disease statuses, including hypertension (yes/no) [26], self-reported diabetes (yes/no), obesity, and high cholesterol. Obesity was defined as body mass index (BMI) ≥ 30 kg/m^2^ [27] while high cholesterol was considered when total cholesterol ≥ 5.18 mmol/L [28]. In stratified and sensitivity analyses, we included age (< 60 years and ≥ 60 years) [29], chronotype (early/late) [30], sleep durations [short (< 7 h), normal (7–8 h), and long (> 9 h)] [9], CKD (yes/no), and diuretic use (yes/no). Further details of the covariates are provided in the Supplementary information section.

### Statistical analysis

Baseline characteristics were reported based on the type of each variable and its distribution. The data were presented as means and standard deviations (continuous variables) and were reported as numbers and percentages (categorical variables). Follow-up duration was determined based on the baseline date (participants attending assessment centers) to the diagnosis of gout, death, or the censoring date (1 March 2022), whichever came first. To evaluate the associations between SW and incident gout, we estimated the hazard ratios (HRs) and 95% confidence intervals (CI) through Cox proportional hazards regression. Proportional hazard assumptions were satisfied after performing the Schoenfeld residuals method. To investigate the association between SW and HUA, we estimated the odds ratios (OR) and 95% CI using logistics regression. Three models were adjusted in our study. Model 1 was adjusted by sex and age. Model 2 was further adjusted by household income, education qualifications, smoking status, drinking frequency, and diet quality. Model 3 was further adjusted by hypertension, diabetes, obesity, and high cholesterol. The approach we implemented for handling missing data was imputing mean values (continuous variables) or using missing indicators (categorical variables) [31].

We also assessed whether current and lifetime SW exacerbated the risk of gout and HUA in individuals genetically predisposed to these conditions. Following prior research [9], we first examined the association between weighted GRS (tertiles) and the risk of gout and HUA. We then evaluated stratified associations by GRS category for individuals with current and lifetime SW data. Interaction P-values were derived using a likelihood ratio test comparing models with and without a cross-product term.

We also performed several sensitivity analyses to examine the robustness of our findings. (1) For gout and HUA, we further adjusted chronotype, sleep duration, and both CKD status and use of diuretics in models 4–6, respectively. (2) We excluded participants diagnosed with gout < 1 year from the baseline date to reassess the estimates. (3) We focused on incident gout cases based solely on hospital inpatient data (ICD-9 and ICD-10 codes); (4) For self-reported gout cases, individuals were excluded if their SUA was < 360 µmol/L and they did not report receiving urate-lowering therapy (allopurinol, probenecid, sulfinpyrazone; febuxostat was not available in the database).

Lastly, the analyses of the associations between current and lifetime SW and gout risk were stratified by sex, age, smoking status, drinking frequency, diet quality score, sleep duration, chronotype, education qualification, household income, and the presence of diabetes, hypertension, obesity, and high cholesterol. Interactions were tested using the aforementioned likelihood ratio test to compare models with and without a cross-product term [9].

Statistical analyses were performed using SPSS software (IBM SPSS Statistics, version 26.0) and R (R version 4.2.3). The forest plots in our study were drawn by the R package “forestploter” (https://CRAN.R-project.org/package=forestploter). The statistical tests in our study were two-tailed. P-values < 0.05 were considered significant.

## Results

### Independent associations of current shift work with incident gout

The baseline characteristics of the included participants are shown in Table 1. Among the 281,500 participants, 82.8% were day workers, and the remaining were shift workers. In comparison to day workers, shift workers were found to have a higher proportion of males, younger individuals, current smokers, and individuals who reported less frequent alcohol consumption or a less healthy diet. Additionally, shift workers tended to report less normal sleep duration, a higher proportion of late chronotype, less college experience, and less high household income. Furthermore, shift workers were more likely to have HUA, obesity, diabetes, hypertension, diuretic use, and less high cholesterol.

**Table 1:** Participants’ characteristics by current shift work exposure (*N* = 281,500)

Baseline characteristics	Day worker	Shift but never/rarely night shifts	Some night shifts	Usual/permanent night shifts
Number	232,948	23,838	13,876	10,838
Male (%)	45.8	46.9	61.2	61.7
Age, mean (SD), years	52.9 (7.1)	52.5 (7.0)	51.1 (6.8)	51.3 (6.8)
Current smoking (%)	9.8	14.0	16.4	17.2
Frequent drinking (%)	73.1	64.8	65.8	61.0
Healthy diet quality (%)	73.3	68.6	66.4	62.8
Normal sleep duration (%)	71.5	65.1	61.9	55.3
Late chronotype (%)	33.8	33.8	35.9	44.2
College or above (%)	40.8	24.6	23.8	15.3
High household income (%)	34.7	18.8	23.5	16.9
Hyperuricemia (%)	10.7	11.4	13.8	14.2
Obese (%)	21.6	27.0	29.2	30.8
Diabetes (%)	3.3	4.3	4.4	5.0
Hypertension (%)	40.7	41.1	42.6	42.6
High cholesterol (%)	69.5	68.3	67.4	65.7
Chronic kidney disease (%)	0.7	0.6	0.5	0.7
Diuretic use (%)	0.5	0.6	0.6	0.6

Participants were included in the analysis of gout. Data was presented as mean (SD) for continuous variables, or percentage for categorical variables

*SD* Standard deviation

During a median follow-up of 13 years, 4,282 incident gout cases were observed. In the current SW analysis, those with shift but never/rarely night shifts, some night shifts, and usual/permanent night shifts were associated with 12.1%, 40.3%, and 16.6% higher gout risks, compared to day workers in the unadjusted model (HR: 1.121, 95% CI: 1.009–1.244, HR: 1.403, 95% CI: 1.243–1.583, HR: 1.166, 95% CI: 1.005–1.352, respectively). In the age- and sex-adjusted model, the HRs of shift but never/rarely night shifts and some night shifts were significant except for usual/permanent night shifts (HR: 1.145, 95% CI: 1.031–1.271, HR: 1.27, 95% CI: 1.125–1.434, HR: 1.056, 95% CI: 0.91–1.225, respectively). After additionally adjusting for socioeconomic and lifestyle factors, only some night shifts were associated with a 22.2% higher gout risk. After further adjustment for chronic disease statuses, this association was attenuated but remained significant (HR:1.151, 95% CI: 1.018–1.301) (Table 2).

**Table 2:** Associations between current, lifetime shift work, and gout risk in the UK Biobank

Work schedules	Incident cases/*N*	Unadjusted		Model 1		Model 2		Model 3	
		HR (95% CI)	*P*	HR (95% CI)	*P*	HR (95% CI)	*P*	HR (95% CI)	*P*
Current shift work									
Day worker	3,422/232,948	Ref	-	Ref	-	Ref	-	Ref	-
Shift but never/rarely night shifts	391/23,838	1.121 (1.009, 1.244)	0.033	1.145 (1.031, 1.271)	0.011	1.101 (0.99, 1.224)	0.076	1.063 (0.956, 1.182)	0.257
Some night shifts	284/13,876	1.403 (1.243, 1.583)	< 0.001	1.27 (1.125, 1.434)	< 0.001	1.222 (1.081, 1.382)	0.001	1.151 (1.018, 1.301)	0.025
Usual/permanent night shifts	185/10,838	1.166 (1.005, 1.352)	0.042	1.056 (0.91, 1.225)	0.473	1.004 (0.864, 1.166)	0.963	0.951 (0.818, 1.105)	0.511
Lifetime shift work Average lifetime night shift frequency									
None	658/56,884	Ref	-	Ref	-	Ref	-	Ref	-
< 3/month	40/2,067	1.676 (1.218, 2.307)	0.002	1.301 (0.945, 1.791)	0.106	1.246 (0.905, 1.717)	0.178	1.199 (0.87, 1.652)	0.267
3–8/month	97/6,845	1.228 (0.992, 1.52)	0.059	1.092 (0.882, 1.353)	0.417	1.041 (0.84, 1.291)	0.712	0.992 (0.8, 1.23)	0.938
> 8/month	165/9,117	1.569 (1.323, 1.861)	< 0.001	1.282 (1.08, 1.521)	0.005	1.197 (1.005, 1.426)	0.044	1.097 (0.92, 1.307)	0.302
Lifetime night shift duration									
None	658/56,884	Ref	-	Ref	-	Ref	-	Ref	-
< 5 years	114/6,432	1.536 (1.259, 1.874)	< 0.001	1.263 (1.035, 1.542)	0.022	1.216 (0.996, 1.485)	0.055	1.164 (0.953, 1.422)	0.137
5–10 years	53/3,979	1.152 (0.871, 1.524)	0.323	1.077 (0.814, 1.425)	0.605	1.015 (0.766, 1.344)	0.919	0.962 (0.726, 1.275)	0.787
> 10 years	135/7,618	1.538 (1.278, 1.851)	< 0.001	1.241 (1.03, 1.494)	0.023	1.15 (0.951, 1.392)	0.149	1.046 (0.864, 1.267)	0.642

Data are presented as hazard ratios (95% confidence interval). Model 1 was adjusted for sex and age. Model 2 was adjusted for the terms in model 1, household income, education qualification, smoking status, drinking frequency, and diet quality. Model 3 was adjusted for terms in model 2, hypertension, diabetes, obesity, and high cholesterol

*CI* Confidence interval, *HR* Hazard ratio

### Independent associations of lifetime shift work with incident gout

Firstly, compared to individuals who never worked night shifts, strong associations were found between lifetime SW frequency and gout risk, especially < 3/month and > 8/month in the unadjusted model (HR: 1.676, 95% CI: 1.218–2.307, HR: 1.569, 95% CI: 1.323–1.861, respectively). In model 3, the HRs (95% CI) across all categories of lifetime SW frequency were considerable but attenuated (HR: 1.199, 95% CI: 0.87–1.652, HR: 0.992, 95% CI: 0.8–1.23, HR: 1.097, 95% CI: 0.92–1.307, respectively). Subsequently, the initial model revealed that < 5 years, 5–10 years, and > 10 years of night shift exposure were associated with 53.6%, 15.2%, and 53.8% higher gout risk, compared to individuals without night shift exposure (HR: 1.536, 95% CI: 1.259–1.874, HR: 1.152, 95% CI: 0.871–1.524, HR: 1.538, 95% CI: 1.278–1.851, respectively). After adjusting all covariates, a 16.4% higher risk was still observed among individuals with < 5 years of night shifts (HR: 1.164, 95% CI: 0.953–1.422) (Table 2). We further excluded individuals diagnosed with gout < 1 year from the baseline and found that those with < 5 years of night shift had a 25.9% higher risk in model 3 (HR: 1.259, 95% CI: 1.027–1.542) (Table S2).

### Independent associations of current and lifetime shift work with hyperuricemia

Participants without SUA data were excluded, and a cross-sectional analysis was performed to examine the relationship between SW and HUA (*N* = 267,892) (Fig. S1). Participants’ characteristics by current SW exposure are summarized in Table S3. The current SW analysis showed that the three SW frequencies increased the likelihood of HUA in the unadjusted model (OR: 1.065, 95% CI: 1.021–1.111, OR: 1.342, 95% CI: 1.277–1.411, OR: 1.357, 95% CI: 1.283–1.435, respectively). In model 3, the effects of some night shifts (OR: 1.109, 95% CI: 1.052–1.169) and usual/permanent night shifts (OR: 1.114, 95% CI: 1.05–1.182) were still significant except for shift but never/rarely night shifts (OR: 0.994, 95% CI: 0.951–1.039). After adjusting multiple covariates, the lifetime SW analysis indicated that < 5 years and > 10 years of night shifts both increased HUA odds (OR: 1.094, 95% CI: 1.004–1.191, OR: 1.071, 95% CI: 0.99–1.158, respectively). Interestingly, an 11.4% lower likelihood was observed among participants with 5–10 years of night shifts (OR: 0.886, 95% CI: 0.79–0.993) (Table S4).

### Joint effects of shift work and genetic risks on incident gout and hyperuricemia

**Fig. 1:**
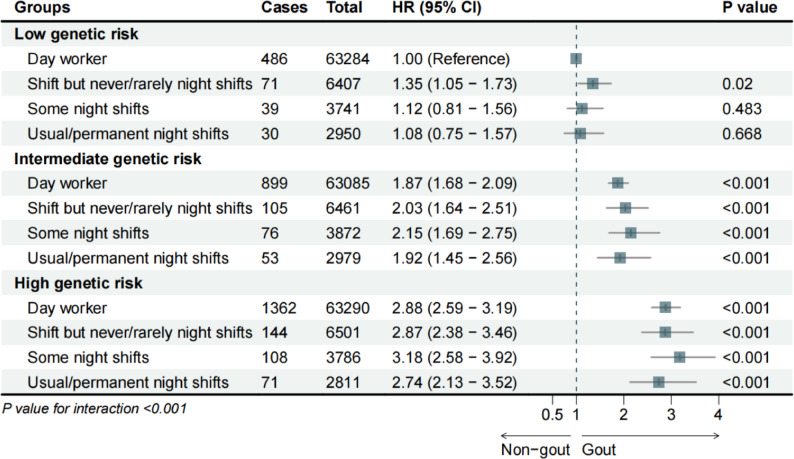
The joint associations of current shift work and genetic risk with incident gout. A multivariable model was adjusted for sex, age, household income, education qualification, smoking status, drinking frequency, diet quality, hypertension, diabetes, obesity, and high cholesterol. CI, confidence interval; HR, hazard ratio

To further investigate the joint effects of the SW and genetic predisposition to gout and HUA, a weighted GRS was developed to assess genetic risk levels, and participants were categorized into low-, intermediate-, and high-GRS groups. As expected, individuals with intermediate and high GRSs had 82.8% and 177.2% higher gout risks compared to those with low GRS, respectively (HR: 1.828, 95% CI: 1.658–2.015, HR: 2.772, 95% CI: 2.529, 3.039) (Table S5). Significant interactions were observed between genetic risk and current or lifetime SW (*P* < 0.001). In the intermediate and high-GRS group, individuals working any night shifts faced notably elevated risks. Notably, individuals with high GRS and some night shifts had a more than three-fold greater risk than those with low GRS and day work (HR:3.18, 95% CI: 2.58–3.92) (Fig. 1). Lifetime SW analysis showed that individuals with low GRS did not show increased vulnerability to incident gout. However, in the high-GRS group, those with < 3 months and < 5 years of night shifts were significantly associated with substantial risk (HR:3.47, 95% CI: 2.10–5.74, HR:3.43, 95% CI: 2.42–4.85, respectively) (Fig. S2).

We also assessed the joint associations of SW schedules and genetic risk with HUA odds. As expected, individuals with intermediate and high GRSs had 76.7% and 208% higher HUA odds compared to those with low GRS, respectively (OR: 1.767, 95% CI: 1.701–1.835, OR: 3.08, 95% CI: 2.972–3.193) (Table S6). In the intermediate- and high-GRS groups, participants following some or usual/permanent night shift schedules were positively associated with elevated HUA odds (Fig. S3). Analysis of lifetime SW exposure revealed that participants with 3–8 months and > 10 years of night shifts had the highest odds (OR:3.59, 95% CI: 3.09–4.17, OR:3.67, 95% CI: 3.19–4.22, respectively) (Fig. S4).

### Subgroup and sensitivity analysis

As shown in Table 3, a significant interaction was observed between smoking status and current SW (P _interaction_ = 0.017). Among previous smokers, some night shifts were associated with a 29.6% elevated risk compared to day workers (HR: 1.296, 95% CI: 1.077–1.559). In current smokers, shift but never/rarely night shifts was linked to a 34% higher gout risk (1.34, 95% CI: 1.018–1.763). Additionally, a significant interaction was found between chronotype and current SW (P _interaction_ = 0.012). Participants reporting an early chronotype had a 27% higher risk of developing gout when having some night shifts (HR: 1.27, 95% CI: 1.078–1.495). On the contrary, among individuals with a late chronotype, usual/permanent night shift was associated with a 23.3% lower risk (HR: 0.767, 95% CI: 0.6–0.982.6.982). In addition, we found that the association between current SW and gout was strengthened in non-obese individuals than in obese ones (P _interaction_ = 0.006). Among non-obese participants, shift but never/rarely night shifts and some night shifts were associated with 25.2% and 22.5% higher risk, respectively (HR: 1.252, 95% CI: 1.091–1.436, HR: 1.225, 95% CI: 1.035–1.45). However, as Tables S7-8 show, no interactions were found between lifetime night shift duration or frequency and gout, except for the interaction between shift duration and sleep duration (an outlier that may distort the interaction effect was identified).

**Table 3:** The stratified associations of current shift work with incident gout in the subgroups

Subgroup	Day workers	Shift but never/rarely night shifts	Some night shifts	Usual/permanent night shifts	*P* for interaction
Sex					0.504
Men	1	1.062 (0.944, 1.194)	1.192 (1.049, 1.356)	0.965 (0.822, 1.132)	
Women	1	1.079 (0.845, 1.376)	0.881 (0.58, 1.339)	0.873 (0.563, 1.354)	
Age					0.389
< 60	1	1.021 (0.896, 1.163)	1.106 (0.96, 1.275)	0.833 (0.696, 0.997)	
≥ 60	1	1.102 (0.92, 1.321)	1.11 (0.869, 1.42)	1.146 (0.875, 1.503)	
Smoking status					0.017
Never	1	1.069 (0.914, 1.25)	1.122 (0.935, 1.346)	1.076 (0.871, 1.329)	
Past	1	0.977 (0.825, 1.157)	1.296 (1.077, 1.559)	0.917 (0.72, 1.166)	
Current	1	1.34 (1.018, 1.763)	0.824 (0.562, 1.209)	0.716 (0.453, 1.129)	
Drinking frequency					0.603
Never	1	1.425 (0.971, 2.09)	0.991 (0.569, 1.728)	0.923 (0.52, 1.638)	
Infrequent	1	1.014 (0.774, 1.329)	1.216 (0.888, 1.665)	1.002 (0.7, 1.434)	
Frequent	1	1.045 (0.926, 1.18)	1.155 (1.007, 1.325)	0.945 (0.795, 1.123)	
Diet quality score					0.661
Poor	1	1.013 (0.702, 1.462)	0.919 (0.591, 1.428)	0.884 (0.545, 1.433)	
Intermediate	1	1.119 (0.92, 1.362)	1.091 (0.861, 1.383)	0.867 (0.652, 1.154)	
Healthy	1	1.054 (0.921, 1.206)	1.22 (1.047, 1.423)	1.037 (0.857, 1.255)	
Sleep duration					0.075
Short	1	1.162 (0.967, 1.396)	0.971 (0.774, 1.218)	0.996 (0.787, 1.26)	
Normal	1	1.04 (0.909, 1.19)	1.241 (1.066, 1.445)	0.921 (0.749, 1.132)	
Long	1	0.779 (0.471, 1.287)	1.287 (0.776, 2.135)	0.899 (0.471, 1.717)	
Chronotype					0.012
Early	1	1.053 (0.91, 1.217)	1.27 (1.078, 1.495)	1.087 (0.87, 1.358)	
Late	1	0.926 (0.763, 1.123)	0.914 (0.727, 1.15)	0.767 (0.6, 0.982)	
Education qualification					0.590
College or above	1	1.212 (0.969, 1.517)	1.131 (0.843, 1.516)	1.255 (0.847, 1.859)	
High school	1	1.053 (0.917, 1.211)	1.148 (0.98, 1.343)	0.836 (0.686, 1.02)	
Less than high school	1	0.966 (0.754, 1.238)	1.215 (0.923, 1.6)	1.114 (0.827, 1.501)	
Household income					0.981
Low	1	1.071 (0.811, 1.415)	1.284 (0.905, 1.821)	1.061 (0.707, 1.59)	
Medium	1	1.013 (0.877, 1.171)	1.138 (0.964, 1.343)	0.964 (0.795, 1.17)	
High	1	1.109 (0.871, 1.411)	1.091 (0.839, 1.417)	0.856 (0.586, 1.249)	
Diabetes					0.593
No	1	1.068 (0.957, 1.192)	1.13 (0.994, 1.284)	0.929 (0.794, 1.088)	
Yes	1	0.993 (0.662, 1.49)	1.392 (0.892, 2.171)	1.3 (0.797, 2.12)	
Hypertension					0.356
No	1	0.939 (0.795, 1.11)	1.163 (0.965, 1.401)	0.936 (0.745, 1.176)	
Yes	1	1.16 (1.012, 1.33)	1.142 (0.97, 1.344)	0.962 (0.788, 1.174)	
Obesity					0.006
No	1	1.252 (1.091, 1.436)	1.225 (1.035, 1.45)	0.949 (0.766, 1.176)	
Yes	1	0.86 (0.728, 1.016)	1.078 (0.902, 1.289)	0.948 (0.768, 1.17)	
High cholesterol					0.960
No	1	1.071 (0.892, 1.285)	1.172 (0.946, 1.452)	1.04 (0.814, 1.33)	
Yes	1	1.062 (0.932, 1.209)	1.143 (0.985, 1.328)	0.911 (0.754, 1.102)	

Data were presented as hazard ratios (95% confidence interval). Data were adjusted for sex, age, smoking status, drinking frequency, diet quality score, education qualification, household income, diabetes, hypertension, obesity, and high cholesterol

To test the robustness of our estimates, we performed various sensitivity analyses. No substantial change was observed in the associations between both current and lifetime SW and incident gout or prevalent HUA (Table S2, S9-12).

## Discussion

In the observational study, based on the large, community-based prospective cohort study with more than 280,000 participants, we examined the association between SW, incident gout, and prevalent HUA, and had the following findings: (1) in the analysis of current SW, participants with some night shifts had a 15.1% higher gout risk, which varied by smoking status, chronotype, and obesity status. The lifetime SW analysis showed that < 3 nights/month and < 5 years of night shifts increased gout risk. Notably, individuals with < 5 years of night shifts  had a 25.9% higher risk after excluding those diagnosed with gout < 1 year; (2) some night shifts, usual/permanent night shifts, and < 5 years of night shifts were significantly associated with 10.9%, 11.4%, and 9.4% higher odds of HUA, respectively; (3) the joint effects of SW and genetic predisposition had a considerable impact on the gout risk. Compared to participants with low genetic risk and day work, those with high genetic risk and some night shifts were associated with a 218% elevated risk of gout.

To our knowledge, the associations of SW with gout or HUA have not been reported previously. Using comprehensive employment data and a large cohort from the UK Biobank, we identified a significant link between SW and the risk of gout or HUA. Specifically, the current SW analysis demonstrated that some night shifts were significantly associated with gout risk after adjusting for various confounds. However, the effect of other SW frequencies on gout risk was not significant (Table 3). In the lifetime SW analysis, the effects of < 3 nights/month and < 5 years of night shifts were more prominent than longer frequencies or durations (Table 3). Several studies have also observed a non-linear association [9, 11, 32], which can be attributed to SW adaptations. Short-term physiological effects of SW (e.g., circadian disruption, poor sleep quality, and duration) may contribute to health and safety risks [33]. However, experienced shift workers may develop coping strategies and resilience over their careers, facilitating long-term adaptation and potentially mitigating adverse effects. Adaptation, however, varies individually, influenced by factors such as age, sex, circadian type, chronotype, and SW experience [34]. Workers unable to adapt may leave their positions for less demanding roles due to health concerns (e.g., gout), supporting the “healthy worker effect” and its explanation of the non-linear relationship between SW and health outcomes [9]. Several factors may explain the attenuation of lifetime SW effects compared to current SW. First, lifetime SW exposure is based on self-reported employment history, which may introduce recall bias and exposure misclassification, potentially weakening exposure–outcome associations [32]. Second, longer tenure and higher qualifications are generally associated with greater job proficiency [35] and lower work intensity [36], leading to an underestimation of exposure intensity over a lifetime. Finally, the small sample size and limited outcome events in long-term follow-ups may reduce statistical power to detect effects [32]. Future cohort studies with objective, longitudinal assessments of SW and larger sample sizes are warranted to clarify its impact on the risk of gout and HUA.

The underlying mechanisms of how SW increased the gout risk or HUA odds were still poorly understood. Several mechanisms we proposed were as follows. Firstly, SW exposure may be associated with an increased risk of sleep disturbances [37]. As a clinical trial indicated, increased levels of catecholamine (including norepinephrine and epinephrine) were observed during sleep deprivation [38], which may induce increased circulating SUA in animal models [39, 40]. In this way, the elevated SUA levels may gradually develop HUA or gout over time. Secondly, SW is often associated with altered lifestyles, including increased alcohol consumption, smoking, reduced physical activity, and poor dietary habits (e.g., low-quality food and excessive calorie intake). These SW-related lifestyles have served as behavioral mechanisms linking SW to adverse health outcomes [8, 41]. Given that SUA levels and gout risk are influenced by unhealthy lifestyle factors [1, 42], it is plausible that the unhealthy lifestyles associated with SW contribute to elevated HUA odds and gout risk. Furthermore, due to inappropriate artificial night light and sunlight exposure, shift workers were often accompanied by circadian rhythm disruption [43, 44], which may activate the NLRP3 inflammasome [45]. Given the NLRP3 inflammasome involved in the gout flare [46], we inferred that its activation caused by SW may contribute to gout development to some extent. Based on these considerations, further studies, such as mediation analysis, could offer valuable insights into potential biological and behavioral mediators linking SW to gout, such as disruptions in sleep patterns, metabolic changes, and behavioral modifications like diet and physical activity. Exploring these mechanisms may identify more targeted preventive interventions for shift workers.

As genetic factors can partially explain the increase of SUA and gout risk [1], GRS for SUA has been constructed to robustly predict the risk of gout and HUA previously [22, 47]. We investigated whether there was a joint association of SW and genetic predisposition with gout or HUA, which has not been reported previously. In our study, Genetic risk significantly modified the associations between SW, gout, and HUA (all P _interaction_ < 0.001) (Fig. 1, S2-4). Several studies have supported the interaction between lifestyle factors and genetic risk in the context of gout and HUA, including healthy lifestyles [48], dietary patterns [49], sleep behaviors [48], etc. However, given that the biological functions of the majority of identified SUA-associated genetic loci are not well comprehended [22], the underlying mechanisms governing the interactions between gene and SW require further elucidation. In the current study, shift workers exhibited a heightened susceptibility to incident gout or prevalent HUA across low-, intermediate-, and high-GRS categories when compared to day workers, which further supported the detrimental effects of SW in isolation. Moreover, this also suggested that the gene-SW interaction exacerbated the deleterious effect of genetic predisposition on gout and HUA. Notably, the impacts of some less strenuous SW schedules were more prominent than the most intense schedule in the high-GRS group, aligning with the “healthy worker effect” described above in our study (Fig. 1, Figs. S2-4). In summary, the joint effects of genetic predisposition and SW exhibit a considerable impact on the gout risk and HUA odds. Given the unmodifiable nature of genetic risk, the selection of a suitable work schedule emerges as a potential preventive measure and is recommended for individuals with elevated genetic risk of gout and HUA.

In stratified analyses, a significant interaction was observed between the current SW and smoking status. Both previous and current smokers were more susceptible to the adverse effects of SW on gout risk (Table 3). This may be due to shift workers have a higher likelihood of smoking behavior [50], which could increase gout risk [51], further exacerbating the detrimental effects of SW. There were strong interactions between the current SW and chronotype (P _interaction_ = 0.012). Participants with early chronotype were more susceptible to developing gout compared to those with late chronotype (Table 3). In line with our findings, an observational study reported that participants with early chronotype were more prone to the impacts of SW, while those with late chronotype were less affected by SW [52, 53]. The potential mechanism may be that participants with early chronotype had difficulties adapting to SW and its related negative effects [54]. Furthermore, the differential metabolites may partially explain why the effects of SW were different from early- and late-chronotype, which required further study for validation [55]. Additionally, a positive interaction between SW and obesity was also observed (P _interaction_ = 0.006). Obesity is a well-established risk factor for gout [2], which may modify the effects of other factors, such as SW. As individuals with obesity already face a heightened baseline risk, non-obese individuals may be more vulnerable to the adverse impacts of SW. Given that non-obese individuals with SW schedules were more prone to developing gout than obese ones (Table 3), non-obese people should make decisions carefully on work schedules to reduce the potential adverse impact of SW in the future.

Our study has several strengths. Firstly, a large sample size of more than 280,000 participants with comprehensive demographic, socioeconomic, lifestyle, and medical conditions information was included in our study, which greatly consolidated the conclusions. In addition, to the best of our knowledge, it was the first study to investigate the association between SW, genetic risk, and risk of gout or HUA. However, our study also has several limitations. Firstly, our observational study only suggests associations. Future research should leverage large-scale GWAS with robust instrumental variables and individual-level data to facilitate comprehensive Mendelian randomization (MR) analyses (e.g., two-sample MR, multivariable MR, and non-linear MR) to establish causality between SW and gout risk. Secondly, both the current and lifetime SW data in our study were mainly based on self-reported employment information by participants, which may introduce potential classification errors [11]. However, the use of standardized questionnaires and clear instructions in the UK Biobank helped minimize reporting bias and improve data quality [56]. Besides, a key limitation of this study is the difficulty in accurately diagnosing gout, as arthrocentesis, the gold standard, is not routinely performed in clinical practice [57]. Additionally, conditions such as calcium pyrophosphate deposition, osteoarthritis, and psoriatic arthritis may mimic gout [1], and the use of the gout diagnostic code as a “working diagnosis” can lead to misclassification. Future cohort studies should integrate clinical, laboratory, and imaging data, leveraging machine learning techniques to enhance diagnostic accuracy and minimize misclassification. Finally, it is important to mention that the individuals involved in our observational research were aged 37–73 years, with a majority being of European descent, particularly White British. Therefore, the data from young workers, different racial backgrounds and diverse regions were insufficient. This limitation may restrict the generalizability and extrapolation of our conclusions.

## Supplementary Information

Supplementary Material 1.

Supplementary Material 2.

## Data Availability

The data that support the findings of this study are available from UK Biobank (https://www.ukbiobank.ac.uk), but restrictions apply to the availability of these data, which were used under license for the current study, and so are not publicly available. Data are, however, available from the authors upon reasonable request and with permission of the UK Biobank.

## Abbreviations

BMI: Body mass index
CI: Confidence interval
CKD: Chronic kidney disease
GRS: Genetic risk score
HR: Hazard ratio
HUA: Hyperuricemia
OR: Odds ratio
SD: Standard deviation
SNP: Single nucleotide polymorphisms
SUA: Serum uric acid
SW: Shift work
