# Supplementary material for: Shift work, genetic risk, and incident gout: a prospective cohort study in the UK Biobank
Source: Arch Public Health. 2026 Mar 17;84:88. doi: 10.1186/s13690-026-01888-1 (PMC13107608; doi:10.1186/s13690-026-01888-1)
Supplement: Supplementary file 2 — Supplementary Material 2. [file 13690_2026_1888_MOESM2_ESM.doc]

**Supplementary Materials for**

**Shift work, genetic risk, and incident gout: A prospective cohort study in the UK Biobank**

*Chuanghai Wu1,2†, Haiyun Zhang3†, Jinjin Xia1†, Baizhao Peng2, Jieyu Chen2, Ming Wang1, Siqi Wu2, Zihao Jiang2,*

*Shuai Ji2, Ying Yang2, Yanting You2, Hiu Yee Kwan4, Zhuhua Sun5, Xiaoshan Zhao1,2*, Yanyan Liu2**

Correspondence to: Prof Xiaoshan, Zhao, zhaoxs@smu.edu.cn

Prof Yanyan, Liu, lyyxi@163.com

Contents

[Supplementary information 3](#__RefHeading___Toc211260050)

[Fig. S1. Flowchart of the participants included in this study. 6](#__RefHeading___Toc211260051)

[Fig. S2. The joint associations of lifetime shift work and genetic risk with incident gout. 7](#__RefHeading___Toc211260052)

[Fig. S3. The joint associations of current shift work and genetic risk with prevalent hyperuricemia. 8](#__RefHeading___Toc211260053)

[Fig. S4. The joint associations of lifetime shift work and genetic risk with prevalent hyperuricemia. 9](#__RefHeading___Toc211260054)

[Table S1. Details of genetic variants used for constructing the genetic risk score for serum urate levels. 10](#__RefHeading___Toc211260055)

[Table S2. HRs (95% CIs) for lifetime shift work with gout ≥ 1 year from the baseline date. 15](#__RefHeading___Toc211260056)

[Table S3. Participants’ characteristics by current shift work exposure (N = 267,892). 17](#__RefHeading___Toc211260057)

[Table S4. Associations between current, lifetime shift work, and hyperuricemia. 18](#__RefHeading___Toc211260058)

[Table S5. HRs (95% CIs) for genetic risk intervals and gout. 19](#__RefHeading___Toc211260059)

[Table S6. ORs (95% CIs) for genetic risk intervals and hyperuricemia. 20](#__RefHeading___Toc211260060)

[Table S7. The stratified associations of lifetime shift work frequency with gout risk in the subgroups. 21](#__RefHeading___Toc211260061)

[Table S8. The stratified associations of lifetime shift work duration with gout risk in the subgroups. 24](#__RefHeading___Toc211260062)

[Table S9. Adjusted HRs (95% CIs) for current and lifetime shift work and incident gout, further adjusted for chronotype (Model 4), sleep duration (Model 5), and chronic kidney disease and diuretic use (Model 6). 27](#__RefHeading___Toc211260063)

[Table S10. Adjusted HRs (95% CIs) for current and lifetime shift work and incident gout, based on hospital inpatient data (ICD-9 and ICD-10 codes). 28](#__RefHeading___Toc211260064)

[Table S11. Adjusted HRs (95% CIs) for current and lifetime shift work and incident gout, excluding self-reported cases if SUA < 360 μmol/L and did not report receiving ULT. 30](#__RefHeading___Toc211260065)

[Table S12. Adjusted ORs (95% CIs) for current and lifetime shift work and prevalent hyperuricemia, further adjusted for chronotype (Model 4), sleep duration (Model 5), and chronic kidney disease and diuretic use (Model 6). 32](#__RefHeading___Toc211260066)

[Table S13. Adjusted odds for lifetime shift work with hyperuricemia after further adjustment for chronotype or sleep duration. 33](#__RefHeading___Toc211260067)

# Supplementary information

Based on previous research, we calculated diet quality scores with moderate modifications [1]. We included four food categories: fruits and vegetables (fresh fruit, dried fruit, cooked vegetables, salad/raw vegetables), oily fish, red meat (beef, lamb/mutton, pork), and processed meat. Each of ≥ 400 g/day of fruits and vegetables, ≥ 1 portion/week of oily fish, ≤ 3 portion/week of red meat, and ≤ 1 portion/week of processed meat was assigned 1 point; otherwise, 0 points were given. The scores for each food category were summed and categorized into five levels (0-4). In this study, scores of 0-1, 2, and 3-4 were classified as poor, intermediate, and healthy diet quality, respectively.

In the UK Biobank, chronotype was determined based on participants’ self-reported chronotype, a method widely used in observational studies [2, 3]. Participants were asked, “Do you consider yourself to be?” Those who responded as “definitely a morning person” or “more of a morning person than an evening person” were classified as having an “early chronotype”, while those who identified as “definitely an evening person” or “more of an evening person than a morning person” were classified as having a “late chronotype”, consistent with prior studies [4, 5].

We calculated estimated glomerular filtration rate (eGFR) using 2021 CKD-EPI-creatinine equation [6]. Chronic kidney disease (CKD) staging was performed in accordance with the Kidney Disease: Improving Global Outcomes (KDIGO) guidelines [7], with CKD defined as an eGFR below 60 ml/min/1.73 m² (i.e., CKD stage 3 or worse). Using self-reported medication data from the UK Biobank, we assessed the use of diuretics, including loop diuretics, thiazide diuretics, and thiazide-like diuretics, which have been linked to an increased risk of gout [8].

**References**

[1] Foster HME, Celis-Morales CA, Nicholl BI, Petermann-Rocha F, Pell JP, Gill JMR, et al. The effect of socioeconomic deprivation on the association between an extended measurement of unhealthy lifestyle factors and health outcomes: a prospective analysis of the UK Biobank cohort. Lancet Public Health 2018; 3:E576-E85.

[2] Vetter C, Dashti HS, Lane JM, Anderson SG, Schernhammer ES, Rutter MK, et al. Night shift work, genetic risk, and type 2 diabetes in the UK Biobank. Diabetes Care 2018; 41:762-69.

[3] Wang N, Sun Y, Zhang H, Wang B, Chen C, Wang Y, et al. Long-term night shift work is associated with the risk of atrial fibrillation and coronary heart disease. Eur Heart J 2021; 42:4180-+.

[4] Fan M, Sun D, Zhou T, Heianza Y, Lv J, Li L, et al. Sleep patterns, genetic susceptibility, and incident cardiovascular disease: a prospective study of 385 292 UK biobank participants. Eur Heart J. 2020;41(11):1182-89.

[5] Li HM, Zhang XR, Liao DQ, Gao J, Qiu CS, Zhong WF, et al. Healthy sleep patterns and risk of hospitalization for infection: a large community-based cohort study. Transl Psychiatry. 2025;15(1):100.

[6] Inker LA, Eneanya ND, Coresh J, Tighiouart H, Wang D, Sang Y, et al. New Creatinine- and Cystatin C-Based Equations to Estimate GFR without Race. N Engl J Med. 2021;385(19):1737-49.

[7] Stevens PE, Levin A. Evaluation and management of chronic kidney disease: synopsis of the kidney disease: improving global outcomes 2012 clinical practice guideline. Ann Intern Med. 2013;158(11):825-30.

[8] Ben Salem C, Slim R, Fathallah N, Hmouda H. Drug-induced hyperuricaemia and gout. Rheumatology (Oxford). 2017;56(5):679-88.

# Fig. S1. Flowchart of the participants included in this study.

**
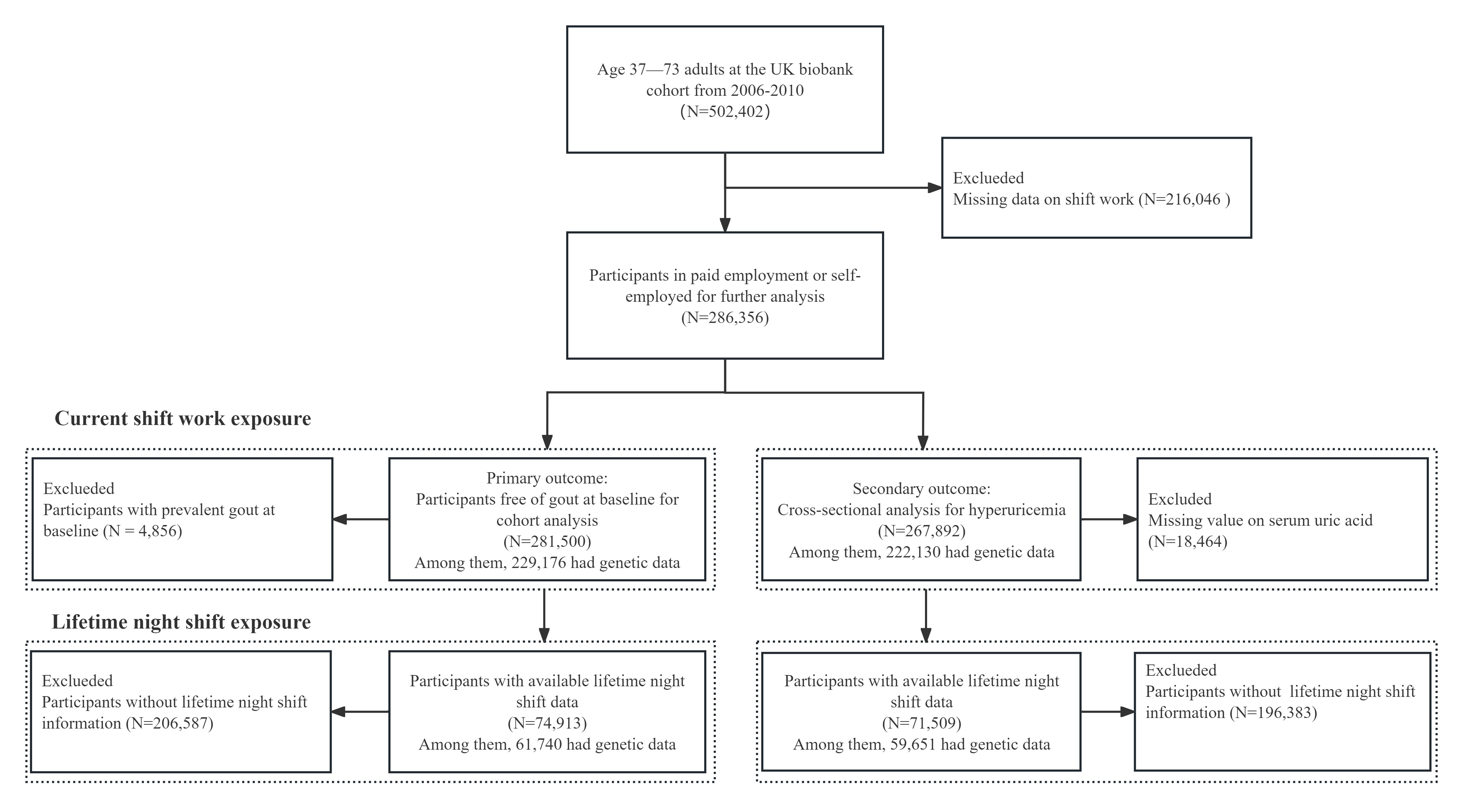
**

# Fig. S2. The joint associations of lifetime shift work and genetic risk with incident gout.

**
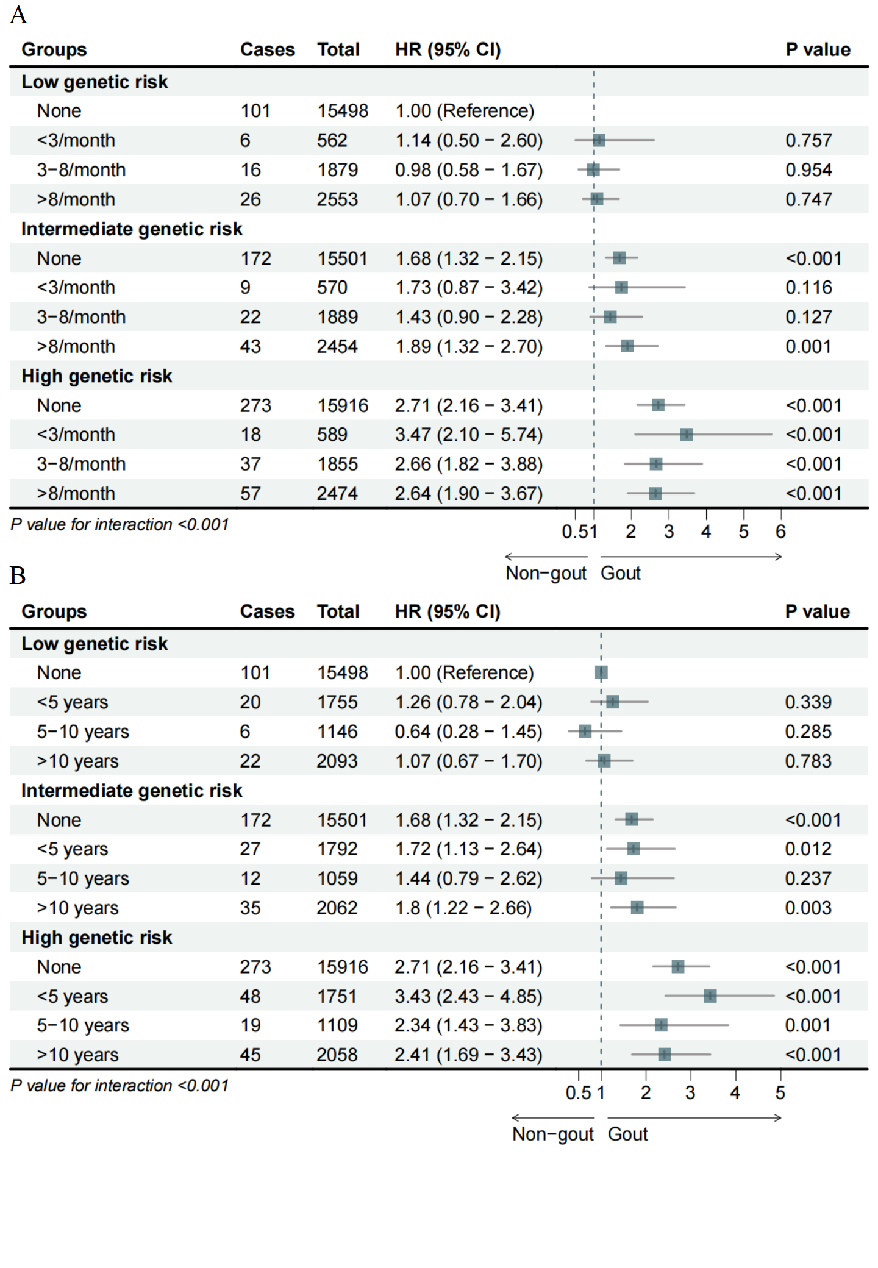
**

Note: A multivariable model was adjusted for sex, age, household income, education qualification, smoking status, drinking frequency, diet quality, hypertension, diabetes, obesity, and high cholesterol. CI, confidence interval; HR, hazard ratio.

# Fig. S3. The joint associations of current shift work and genetic risk with prevalent hyperuricemia.

**
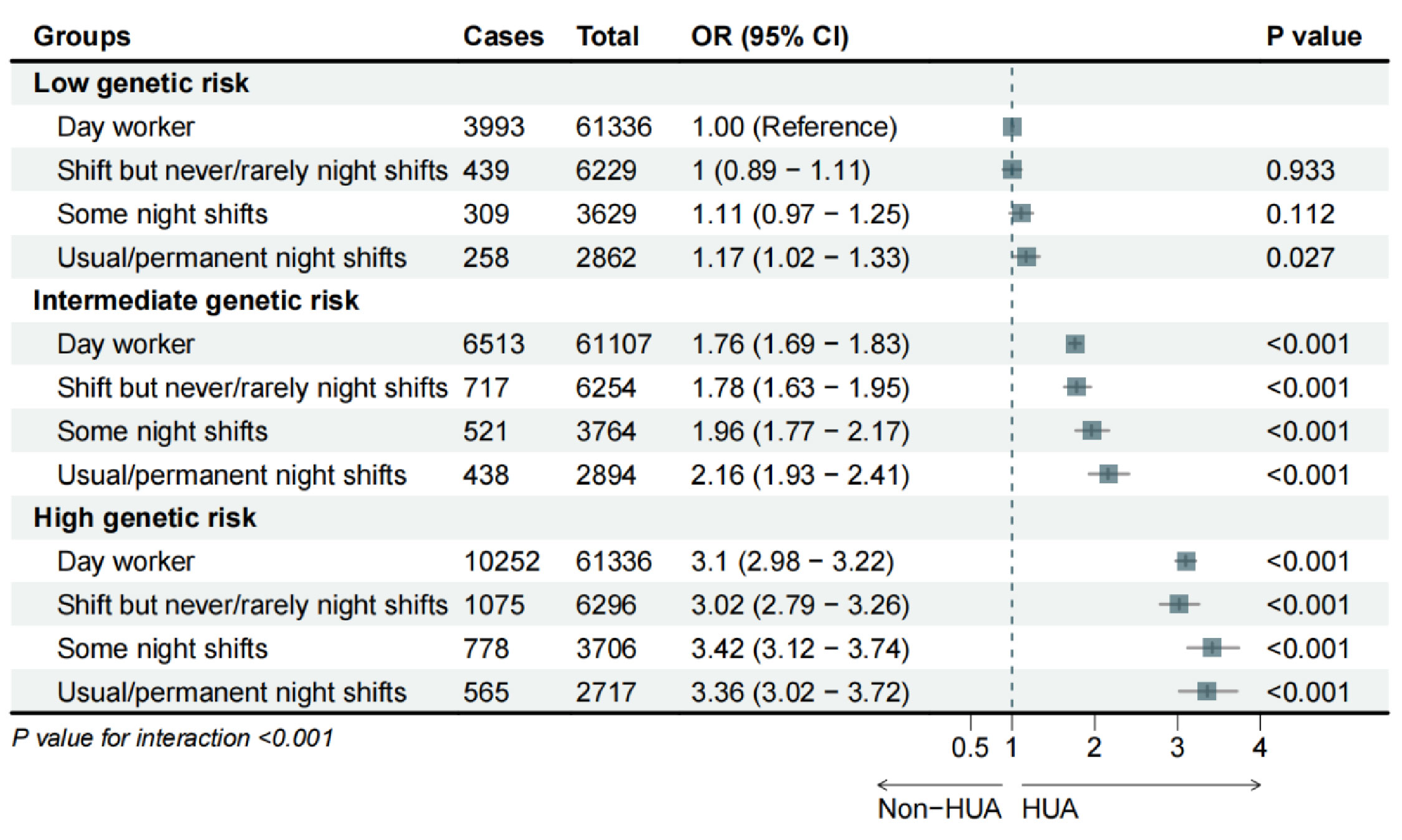
**

Note: A multivariable model was adjusted for sex, age, household income, education qualification, smoking status, drinking frequency, diet quality, hypertension, diabetes, obesity, and high cholesterol. CI, confidence interval; HUA, hyperuricemia; OR, odds ratio.

# Fig. S4. The joint associations of lifetime shift work and genetic risk with prevalent hyperuricemia.

**
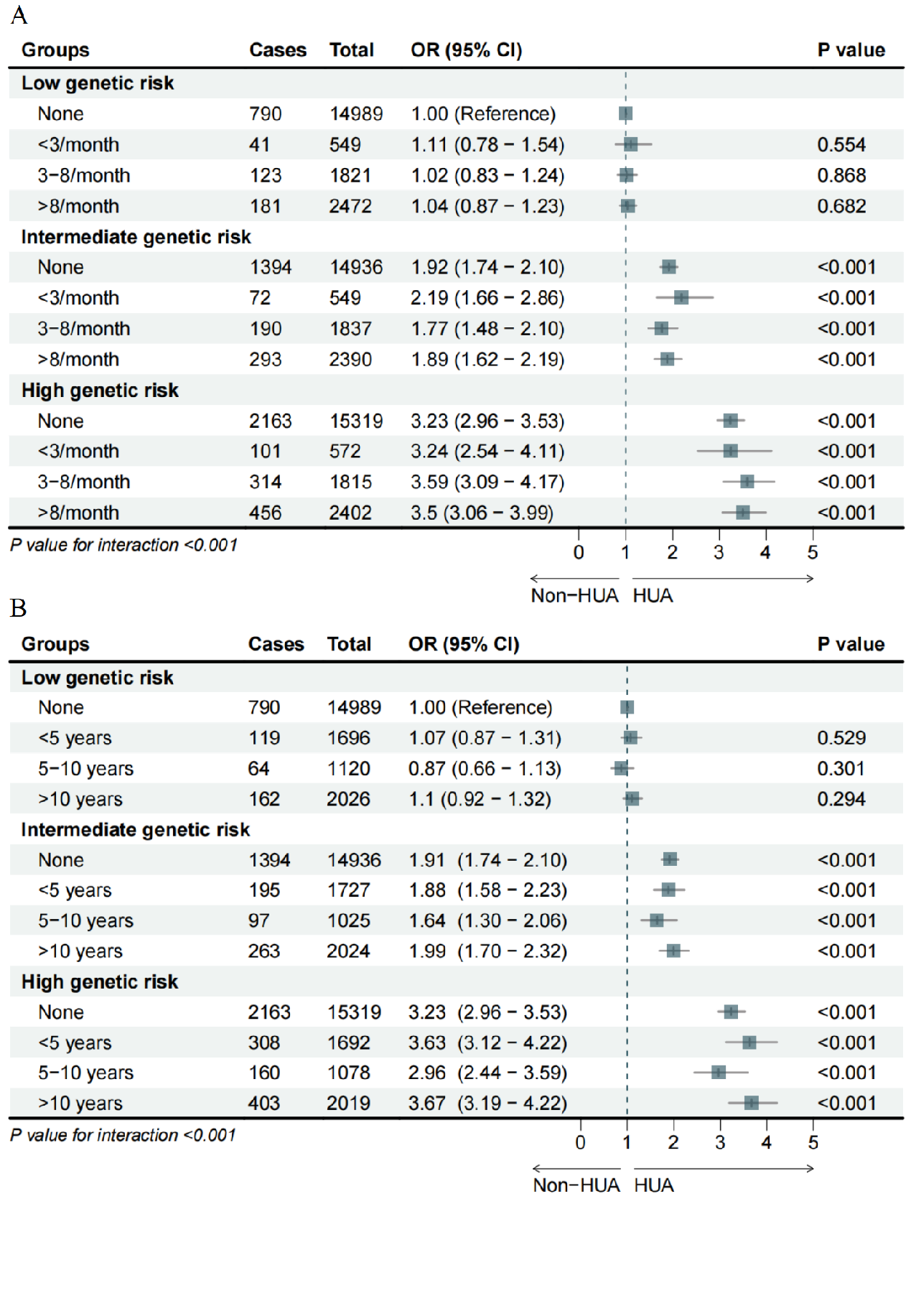
**

Note: A multivariable model was adjusted for sex, age, household income, education qualification, smoking status, drinking frequency, diet quality, hypertension, diabetes, obesity, and high cholesterol. CI, confidence interval; HUA, hyperuricemia; OR, odds ratio.

# Table S1. Details of genetic variants used for constructing the genetic risk score for serum urate levels.

| **Region** | **Chromosome** | **Independent**  **SNP** | **Closest**  **gene** | **Coded**  **allele** | **Coded allele**  **frequency** | **Effect** | **SE** | **Meta-analysis**  **P-value** |
| --- | --- | --- | --- | --- | --- | --- | --- | --- |
| 1 | 1 | rs2480712 | *SKI* | C | 0.662 | 0.024 | 0.004 | 6.25E-09 |
| 2 | 1 | rs4646068 | *CASP9* | T | 0.692 | 0.024 | 0.004 | 7.71E-09 |
| 3 | 1 | rs79598313 | *KDF1* | T | 0.026 | 0.100 | 0.013 | 9.22E-15 |
| 4 | 1 | rs141990161 | *HAO2* | T | 0.985 | 0.133 | 0.023 | 1.59E-08 |
| 5 | 1 | rs139428292 | *NBPF20* | A | 0.027 | -0.073 | 0.013 | 4.91E-08 |
| 5 | 1 | rs10910845 | *NBPF20* | A | 0.469 | 0.058 | 0.004 | 1.50E-51 |
| 6 | 1 | rs11204701 | *GOLPH3L* | A | 0.779 | -0.036 | 0.005 | 1.05E-14 |
| 7 | 1 | rs2070803 | *TRIM46* | A | 0.578 | 0.053 | 0.004 | 4.09E-41 |
| 8 | 1 | rs2760215 | *LOC100422212* | T | 0.503 | -0.025 | 0.004 | 5.81E-11 |
| 9 | 1 | rs12037861 | *HLX-AS1* | A | 0.704 | 0.023 | 0.004 | 3.39E-08 |
| 10 | 2 | rs2867112 | *TMEM18* | T | 0.830 | 0.035 | 0.005 | 9.84E-12 |
| 11 | 2 | rs72782806 | *DDX1* | A | 0.260 | 0.025 | 0.004 | 8.12E-09 |
| 12 | 2 | rs1260326 | *GCKR* | T | 0.398 | 0.070 | 0.004 | 4.61E-69 |
| 13 | 2 | rs12472381 | *LINC01122* | A | 0.390 | 0.022 | 0.004 | 1.80E-08 |
| 14 | 2 | rs12987661 | *AAK1* | T | 0.866 | 0.041 | 0.006 | 1.44E-12 |
| 15 | 2 | rs759219 | *ATP6V1B1* | T | 0.439 | -0.022 | 0.004 | 7.93E-09 |
| 16 | 2 | rs17050272 | *LINC01101* | A | 0.421 | 0.032 | 0.004 | 1.57E-15 |
| 17 | 2 | rs11683692 | *TEX41* | T | 0.944 | -0.048 | 0.008 | 1.32E-08 |
| 18 | 2 | rs1234413 | *MBD5* | T | 0.442 | -0.022 | 0.004 | 7.08E-09 |
| 19 | 2 | rs9287911 | *LRP2* | A | 0.250 | 0.038 | 0.004 | 1.13E-17 |
| 20 | 2 | rs187355703 | *HOXD8* | C | 0.975 | -0.086 | 0.013 | 2.70E-11 |
| 21 | 2 | rs1047891 | *CPS1* | A | 0.311 | -0.024 | 0.004 | 2.09E-08 |
| 22 | 2 | rs9288447 | *ERBB4* | T | 0.546 | -0.023 | 0.004 | 3.27E-09 |
| 23 | 3 | rs2581817 | *SFMBT1* | C | 0.420 | 0.048 | 0.004 | 4.87E-35 |
| 24 | 3 | rs11128111 | *ARL6IP5* | T | 0.480 | -0.021 | 0.004 | 4.64E-08 |
| 25 | 3 | rs7640441 | *ZNF148* | A | 0.246 | -0.028 | 0.005 | 1.26E-09 |
| 25 | 3 | rs11718633 | *KLF15* | T | 0.198 | -0.028 | 0.005 | 7.05E-09 |
| 26 | 3 | rs80120242 | *DNAJC13* | A | 0.947 | -0.062 | 0.010 | 1.87E-09 |
| 27 | 3 | rs62294340 | *MECOM* | A | 0.364 | -0.022 | 0.004 | 5.00E-08 |
| 28 | 4 | rs6825187 | *SLC2A9* | T | 0.357 | 0.187 | 0.004 | 0 |
| 28 | 4 | rs62286563 | *WDR1* | T | 0.979 | -0.103 | 0.015 | 1.16E-12 |
| 28 | 4 | rs10017305 | *ZNF518B* | T | 0.709 | 0.214 | 0.005 | 0 |
| 28 | 4 | rs73224492 | *ZNF518B* | A | 0.875 | -0.094 | 0.006 | 9.00E-59 |
| 29 | 4 | rs98270 | *NIPAL1* | A | 0.362 | 0.022 | 0.004 | 4.21E-08 |
| 30 | 4 | rs10857147 | *FGF5* | A | 0.713 | 0.024 | 0.004 | 2.21E-08 |
| 31 | 4 | rs188917216 | *SPP1* | A | 0.988 | -0.143 | 0.023 | 6.44E-10 |
| 31 | 4 | rs1481012 | *ABCG2* | A | 0.889 | -0.249 | 0.006 | 0 |
| 31 | 4 | rs2622629 | *ABCG2* | T | 0.636 | -0.057 | 0.004 | 8.42E-44 |
| 32 | 4 | rs1440411 | *USP38* | T | 0.571 | -0.028 | 0.004 | 1.08E-12 |
| 33 | 5 | rs455213 | *RAI14* | T | 0.543 | -0.027 | 0.004 | 6.05E-12 |
| 34 | 5 | rs10942549 | *TMEM171* | C | 0.312 | -0.042 | 0.004 | 1.64E-22 |
| 35 | 5 | rs76004499 | *NSD1* | C | 0.972 | -0.074 | 0.013 | 3.27E-08 |
| 36 | 6 | rs12530084 | *RREB1* | T | 0.220 | 0.066 | 0.005 | 9.55E-48 |
| 37 | 6 | rs1359232 | *SLC17A1* | A | 0.466 | -0.091 | 0.004 | 4.12E-126 |
| 37 | 6 | rs198851 | *HIST1H4C* | T | 0.144 | 0.039 | 0.005 | 5.80E-13 |
| 38 | 6 | rs742493 | *UNC5CL* | T | 0.881 | 0.039 | 0.006 | 9.79E-11 |
| 39 | 6 | rs1574430 | *SLC22A7* | A | 0.405 | 0.029 | 0.004 | 2.77E-14 |
| 39 | 6 | rs10223666 | *VEGFA* | C | 0.704 | 0.046 | 0.004 | 6.62E-28 |
| 40 | 6 | rs4897160 | *NCOA7* | A | 0.483 | 0.030 | 0.004 | 1.96E-14 |
| 41 | 7 | rs62435145 | *UNCX* | T | 0.689 | 0.042 | 0.005 | 2.36E-16 |
| 42 | 7 | rs13226650 | *MLXIPL* | A | 0.809 | 0.049 | 0.005 | 1.35E-23 |
| 43 | 7 | rs11551890 | *TECPR1* | A | 0.509 | 0.023 | 0.004 | 2.40E-08 |
| 44 | 7 | rs10480300 | *PRKAG2* | T | 0.276 | 0.030 | 0.004 | 4.26E-12 |
| 45 | 8 | rs34861762 | *STC1* | T | 0.419 | 0.034 | 0.004 | 3.50E-19 |
| 46 | 8 | rs2466077 | *NRG1* | T | 0.533 | -0.022 | 0.004 | 1.78E-08 |
| 47 | 8 | rs2943539 | *HNF4G* | T | 0.475 | 0.041 | 0.004 | 6.42E-28 |
| 48 | 8 | rs10956924 | *ESRP1* | T | 0.279 | -0.024 | 0.004 | 1.79E-08 |
| 49 | 9 | rs10971420 | *B4GALT1* | T | 0.688 | 0.031 | 0.004 | 4.14E-14 |
| 50 | 9 | rs56106601 | *FAM102A* | A | 0.946 | 0.061 | 0.009 | 2.68E-11 |
| 51 | 10 | rs74440730 | *CUBN* | A | 0.892 | -0.037 | 0.006 | 2.22E-09 |
| 52 | 10 | rs10994860 | *A1CF* | T | 0.180 | 0.064 | 0.005 | 1.31E-36 |
| 53 | 10 | rs1649078 | *BICC1* | A | 0.480 | -0.039 | 0.004 | 4.09E-19 |
| 54 | 10 | rs1171617 | *SLC16A9* | T | 0.768 | 0.079 | 0.005 | 1.81E-66 |
| 55 | 10 | rs9420446 | *FAM35A* | T | 0.137 | -0.038 | 0.006 | 1.13E-11 |
| 56 | 10 | rs35198068 | *TCF7L2* | T | 0.706 | 0.025 | 0.004 | 5.85E-09 |
| 57 | 11 | rs35506085 | *IGF2* | A | 0.189 | -0.029 | 0.005 | 1.50E-08 |
| 58 | 11 | rs148185902 | *MPPED2* | A | 0.012 | 0.123 | 0.023 | 5.76E-08 |
| 58 | 11 | rs3925584 | *DCDC1* | T | 0.552 | 0.030 | 0.004 | 1.66E-15 |
| 59 | 11 | rs71456318 | *SLC22A11* | A | 0.484 | 0.079 | 0.004 | 4.41E-92 |
| 59 | 11 | rs2022051 | *SLC22A12* | A | 0.793 | -0.070 | 0.005 | 5.13E-48 |
| 60 | 11 | rs10896028 | *RELA* | A | 0.645 | -0.048 | 0.004 | 4.10E-33 |
| 61 | 11 | rs10892354 | *USP2* | T | 0.380 | 0.030 | 0.004 | 2.60E-13 |
| 62 | 12 | rs7303595 | *RERG* | A | 0.336 | 0.025 | 0.004 | 7.05E-10 |
| 63 | 12 | rs7315236 | *LOC105369971* | T | 0.357 | 0.029 | 0.004 | 1.91E-13 |
| 64 | 12 | rs12313306 | *R3HDM2* | T | 0.246 | -0.076 | 0.004 | 6.74E-65 |
| 65 | 12 | rs10774625 | *ATXN2* | A | 0.483 | 0.032 | 0.004 | 5.54E-17 |
| 66 | 12 | rs1800574 | *HNF1A* | T | 0.031 | -0.081 | 0.012 | 2.84E-12 |
| 67 | 12 | rs28530689 | *LOC100506691* | A | 0.512 | 0.032 | 0.004 | 1.27E-16 |
| 68 | 12 | rs12423664 | *FBRSL1* | A | 0.152 | 0.042 | 0.006 | 1.75E-13 |
| 69 | 13 | rs7986094 | *HMGB1* | A | 0.302 | -0.024 | 0.004 | 1.74E-08 |
| 70 | 13 | rs626277 | *DACH1* | A | 0.594 | 0.026 | 0.004 | 2.69E-11 |
| 71 | 14 | rs861536 | *KLC1* | A | 0.621 | 0.024 | 0.004 | 2.16E-09 |
| 72 | 15 | rs1478604 | *THBS1* | T | 0.706 | -0.026 | 0.004 | 4.49E-10 |
| 73 | 15 | rs2929508 | *MYO9A* | A | 0.261 | -0.029 | 0.005 | 3.65E-09 |
| 74 | 15 | rs8040109 | *NEO1* | A | 0.707 | 0.025 | 0.004 | 5.85E-09 |
| 75 | 15 | rs2472297 | *CYP1A1* | T | 0.249 | -0.028 | 0.005 | 1.50E-08 |
| 76 | 15 | rs57737646 | *NRG4* | T | 0.025 | -0.094 | 0.012 | 5.40E-14 |
| 76 | 15 | rs10851885 | *NRG4* | A | 0.756 | -0.054 | 0.005 | 4.16E-32 |
| 77 | 15 | rs55781567 | *CHRNA5* | C | 0.655 | 0.023 | 0.004 | 1.11E-08 |
| 78 | 15 | rs12908437 | *IGF1R* | T | 0.376 | 0.046 | 0.004 | 1.56E-30 |
| 79 | 16 | rs4997081 | *UMOD* | C | 0.196 | -0.030 | 0.005 | 4.18E-10 |
| 80 | 16 | rs8050136 | *FTO* | A | 0.403 | 0.025 | 0.004 | 2.34E-10 |
| 81 | 16 | rs62052820 | *MIR1538* | A | 0.212 | 0.041 | 0.005 | 2.81E-18 |
| 82 | 16 | rs4788815 | *TAT* | A | 0.357 | -0.026 | 0.004 | 7.44E-11 |
| 83 | 16 | rs57652769 | *MAFTRR* | T | 0.309 | -0.036 | 0.004 | 8.56E-18 |
| 83 | 16 | rs9925837 | *LINC01229* | A | 0.845 | -0.042 | 0.005 | 5.85E-15 |
| 84 | 16 | rs11644696 | *CMIP* | A | 0.477 | 0.022 | 0.004 | 1.41E-08 |
| 85 | 17 | rs2453580 | *SLC47A1* | T | 0.598 | 0.025 | 0.004 | 7.01E-10 |
| 86 | 17 | rs3794748 | *HLF* | A | 0.409 | 0.038 | 0.004 | 1.38E-21 |
| 87 | 17 | rs9895661 | *BCAS3* | T | 0.817 | 0.050 | 0.005 | 7.23E-23 |
| 88 | 18 | rs11663816 | *MC4R* | T | 0.730 | -0.030 | 0.004 | 1.40E-12 |
| 89 | 19 | rs57070985 | *KDM4B* | A | 0.646 | 0.029 | 0.004 | 2.04E-12 |
| 90 | 19 | rs10405423 | *INSR* | A | 0.663 | 0.039 | 0.004 | 1.07E-20 |
| 91 | 19 | rs4808762 | *PDE4C* | T | 0.720 | -0.024 | 0.004 | 1.36E-08 |
| 92 | 19 | rs2868194 | *SLC7A9* | T | 0.408 | -0.027 | 0.004 | 8.90E-12 |
| 93 | 19 | rs35396326 | *NECTIN2* | C | 0.704 | 0.025 | 0.004 | 2.31E-08 |
| 94 | 19 | rs62128132 | *CPT1C* | T | 0.966 | -0.118 | 0.015 | 1.32E-15 |
| 94 | 19 | rs10414501 | *TSKS* | C | 0.957 | -0.125 | 0.018 | 1.56E-12 |
| 95 | 20 | rs7267595 | *JAG1* | A | 0.510 | 0.023 | 0.004 | 3.15E-09 |
| 96 | 20 | rs6119510 | *TP53INP2* | T | 0.596 | -0.023 | 0.004 | 3.20E-09 |
| 97 | 20 | rs142773928 | *HNF4A* | A | 0.166 | 0.032 | 0.005 | 3.77E-09 |
| 97 | 20 | rs1800961 | *HNF4A* | T | 0.034 | -0.076 | 0.012 | 1.63E-10 |
| 98 | 21 | rs219781 | *CLDN14* | T | 0.246 | -0.025 | 0.004 | 1.56E-08 |
| 99 | 22 | rs12485100 | *PNPLA3* | T | 0.173 | -0.033 | 0.005 | 2.44E-10 |

Note: SE, standard error; SNP, single nucleotide polymorphism. Sources: Tin, A., et al. (2019). Target genes, variants, tissues and transcriptional pathways influencing human serum urate levels. Nat Genet 51(10): 1459-1474. Doi: 10.1038/s41588-019-0504-x.

# Table S2. HRs (95% CIs) for lifetime shift work with gout ≥ 1 year from the baseline date.

| **Shift work schedule** | **Incident cases/N** | **Unadjusted** | | **Model 1** | | **Model 2** | | **Model 3** | |
| --- | --- | --- | --- | --- | --- | --- | --- | --- | --- |
|  |  | **HR (95% CI)** | **P** | **HR (95% CI)** | **P** | **HR (95% CI)** | **P** | **HR (95% CI)** | **P** |
| **Current shift work** |  |  |  |  |  |  |  |  |  |
| Day worker | 3,173/232,699 | Ref | - | Ref | - | Ref | - | Ref | - |
| Shift but never/rarely night shifts | 371/23,818 | 1.147 (1.03, 1.278) | 0.012 | 1.174 (1.054, 1.307) | 0.004 | 1.125 (1.009, 1.254) | 0.034 | 1.086 (0.974, 1.211) | 0.136 |
| Some night shifts | 261/13,853 | 1.391 (1.226, 1.578) | <0.001 | 1.265 (1.115, 1.436) | <0.001 | 1.214 (1.068, 1.379) | 0.003 | 1.143 (1.005, 1.298) | 0.041 |
| Usual/permanent night shifts | 175/10,828 | 1.189 (1.022, 1.385) | 0.025 | 1.083 (0.929, 1.261) | 0.308 | 1.024 (0.878, 1.195) | 0.759 | 0.971 (0.832, 1.133) | 0.706 |
| **Lifetime night shift work** |  |  | |  | |  | |  | |
| **Average lifetime night shift frequency** |  |  |  |  |  |  |  |  |  |
| None | 598/56,824 | Ref | - | Ref | - | Ref | - | Ref | - |
| <3/month | 40/2,067 | 1.845 (1.34, 2.541) | <0.001 | 1.435 (1.042, 1.977) | 0.027 | 1.375 (0.997, 1.896) | 0.052 | 1.322 (0.959, 1.824) | 0.089 |
| 3-8/month | 92/6,840 | 1.282 (1.029, 1.597) | 0.027 | 1.145 (0.919, 1.427) | 0.228 | 1.089 (0.873, 1.359) | 0.448 | 1.035 (0.829, 1.292) | 0.761 |
| >8/month | 156/9,108 | 1.633 (1.369, 1.947) | <0.001 | 1.339 (1.122, 1.598) | 0.001 | 1.245 (1.039, 1.491) | 0.017 | 1.14 (0.951, 1.366) | 0.157 |
| **Lifetime duration of night shift work** |  |  |  |  |  |  |  |  |  |
| None | 598/56,824 | Ref | - | Ref | - | Ref | - | Ref | - |
| <5 years | 112/6,430 | 1.661 (1.357, 2.032) | <0.001 | 1.37 (1.119, 1.677) | 0.002 | 1.317 (1.075, 1.614) | 0.008 | 1.259 (1.027, 1.542) | 0.027 |
| 5-10 years | 49/3,975 | 1.172 (0.876, 1.568) | 0.287 | 1.099 (0.821, 1.47) | 0.527 | 1.032 (0.77, 1.384) | 0.832 | 0.978 (0.73, 1.311) | 0.882 |
| >10 years | 127/7,610 | 1.593 (1.315, 1.929) | <0.001 | 1.291 (1.065, 1.564) | 0.009 | 1.19 (0.977, 1.449) | 0.083 | 1.081 (0.887, 1.318) | 0.438 |

Note: Data was presented as hazard ratios (95% confidence interval). Model 1 was adjusted for sex and age. Model 2 was adjusted for the terms in model 1, household income, education qualification, smoking status, drinking frequency, and diet quality. Model 3 was adjusted for terms in model 2, hypertension, diabetes, obesity, and high cholesterol. CI, confidence interval; HR, hazard ratio.

# Table S3. Participants’ characteristics by current shift work exposure (N = 267,892).

| **Baseline**  **characteristics** | **Day worker** | **Shift but never/rarely**  **night shifts** | **Some night shifts** | **Usual/permanent**  **night shifts** |
| --- | --- | --- | --- | --- |
|
|
| Number | 221,620 | 22,681 | 13,289 | 10,302 |
| Male (%) | 46.8 | 47.8 | 62.3 | 62.7 |
| Age, mean (SD), years | 53.0 (7.1) | 52.5 (7.0) | 51.2 (6.9) | 51.3 (6.8) |
| Current smoking (%) | 9.7 | 13.9 | 16.4 | 17.2 |
| Frequent drinking (%) | 73.4 | 65.2 | 66.4 | 61.5 |
| Healthy diet quality (%) | 73.1 | 68.6 | 66.4 | 62.5 |
| Normal sleep duration (%) | 71.5 | 65.2 | 61.8 | 55.6 |
| Late chronotype (%) | 34.0 | 34.1 | 36.3 | 44.7 |
| College or above (%) | 41.0 | 24.6 | 23.7 | 15.3 |
| High household income (%) | 35.2 | 19.0 | 23.7 | 17.2 |
| Hyperuricemia (%) | 11.3 | 12.0 | 14.6 | 14.8 |
| Obese (%) | 22.0 | 27.4 | 29.7 | 31.1 |
| Diabetes (%) | 3.3 | 4.4 | 4.6 | 5.2 |
| Hypertension (%) | 41.2 | 41.5 | 43.2 | 43.1 |
| High cholesterol (%) | 67.3 | 66.1 | 64.9 | 63.0 |
| Chronic kidney disease (%) | 0.4 | 0.5 | 0.4 | 0.5 |
| Diuretic use (%) | 0.5 | 0.6 | 0.6 | 0.6 |

Note: Participants were included for the analysis of hyperuricemia. Data was presented as mean (SD) for continuous variables, or percentage for categorical variables. SD, standard deviation.

# Table S4. Associations between current, lifetime shift work, and hyperuricemia.

| **Work schedules** | **Prevalent cases/N** | **Unadjusted** | | **Model 1** | | **Model 2** | | **Model 3** | |
| --- | --- | --- | --- | --- | --- | --- | --- | --- | --- |
|  |  | **OR (95% CI)** | **P** | **OR (95% CI)** | **P** | **OR (95% CI)** | **P** | **OR (95% CI)** | **P** |
| **Current night shift work** |  |  |  |  |  |  |  |  |  |
| Day worker | 25,086/221,620 | Ref | - | Ref | - | Ref | - | Ref | - |
| Shift but never/rarely night shifts | 2,715/22,681 | 1.065 (1.021, 1.111) | 0.003 | 1.072 (1.027, 1.118) | 0.002 | 1.045 (1.001, 1.091) | 0.047 | 0.994 (0.951, 1.039) | 0.79 |
| Some night shifts | 1,944/13,289 | 1.342 (1.277, 1.411) | <0.001 | 1.233 (1.172, 1.297) | <0.001 | 1.203 (1.143, 1.266) | <0.001 | 1.109 (1.052, 1.169) | <0.001 |
| Usual/permanent night shifts | 1,521/10,302 | 1.357 (1.283, 1.435) | <0.001 | 1.242 (1.173, 1.315) | <0.001 | 1.195 (1.128, 1.266) | <0.001 | 1.114 (1.05, 1.182) | <0.001 |
| **Lifetime night shift work** |  |  |  |  |  |  |  |  |  |
| **Average lifetime night shift frequency** |  |  |  |  |  |  |  |  |  |
| None | 5,226/54,199 | Ref | - | Ref | - | Ref | - | Ref | - |
| <3/month | 254/1,978 | 1.381 (1.207, 1.58) | <0.001 | 1.186 (1.034, 1.359) | 0.015 | 1.145 (0.998, 1.314) | 0.053 | 1.084 (0.94, 1.25) | 0.265 |
| 3-8/month | 767/6,570 | 1.239 (1.143, 1.342) | <0.001 | 1.149 (1.058, 1.247) | 0.001 | 1.106 (1.019, 1.201) | 0.017 | 1.029 (0.944, 1.12) | 0.517 |
| >8/month | 1,141/8,762 | 1.403 (1.31, 1.502) | <0.001 | 1.234 (1.151, 1.324) | <0.001 | 1.161 (1.082, 1.247) | <0.001 | 1.036 (0.963, 1.116) | 0.343 |
| **Lifetime night shift duration** |  |  |  |  |  |  |  |  |  |
| None | 5,226/54,199 | Ref | - | Ref | - | Ref | - | Ref | - |
| <5 years | 773/6,142 | 1.349 (1.245, 1.462) | <0.001 | 1.2 (1.106, 1.303) | <0.001 | 1.165 (1.073, 1.265) | <0.001 | 1.094 (1.004, 1.191) | 0.04 |
| 5-10 years | 386/3,831 | 1.05 (0.942, 1.171) | 0.38 | 1 (0.896, 1.117) | 0.997 | 0.96 (0.859, 1.072) | 0.467 | 0.886 (0.79, 0.993) | 0.038 |
| >10 years | 1,003/7,337 | 1.484 (1.38, 1.595) | <0.001 | 1.294 (1.201, 1.393) | <0.001 | 1.217 (1.128, 1.313) | <0.001 | 1.071 (0.99, 1.158) | 0.088 |

Note: Data was presented as odds ratios (95% confidence interval). Model 1 was adjusted for sex and age. Model 2 was adjusted for the terms in model 1, household income, education qualification, smoking status, drinking frequency, and diet quality. Model 3 was adjusted for terms in model 2, hypertension, diabetes, obesity, and high cholesterol. CI, confidence interval; HUA, hyperuricemia; OR, odds ratio.

# Table S5. HRs (95% CIs) for genetic risk intervals and gout.

| **Genetic risk** | **Incident cases/N** | **Model 1** | | **Model 2** | | **Model 3** | |
| --- | --- | --- | --- | --- | --- | --- | --- |
|  |  | **HR (95% CI)** | **P** | **HR (95% CI)** | **P** | **HR (95% CI)** | **P** |
| Low genetic risk | 626/76,382 | Ref | - | Ref | - | Ref | - |
| Intermediate genetic risk | 1,133/76,397 | 1.826 (1.656, 2.013) | <0.001 | 1.83 (1.66, 2.017) | <0.001 | 1.828 (1.658, 2.015) | <0.001 |
| High genetic risk | 1,685/76,388 | 2.758 (2.516, 3.023) | <0.001 | 2.762 (2.52, 3.027) | <0.001 | 2.772 (2.529, 3.039) | <0.001 |

Note: Data was presented as hazard ratios (95% confidence interval). Model 1 was adjusted for sex and age. Model 2 was adjusted for the terms in model 1, household income, education qualification, smoking status, drinking frequency, and diet quality. Model 3 was adjusted for terms in model 2, hypertension, diabetes, obesity, and high cholesterol. CI, confidence interval; HR, hazard ratio.

# Table S6. ORs (95% CIs) for genetic risk intervals and hyperuricemia.

| **Genetic risk** | **Incident cases/N** | **Model 1** | | **Model 2** | | **Model 3** | |
| --- | --- | --- | --- | --- | --- | --- | --- |
|  |  | **OR (95% CI)** | **P** | **OR (95% CI)** | **P** | **OR (95% CI)** | **P** |
| Low genetic risk | 4,999/74,056 | Ref | - | Ref | - | Ref | - |
| Intermediate genetic risk | 8,189/74,019 | 1.734 (1.671, 1.8) | <0.001 | 1.74 (1.676, 1.806) | <0.001 | 1.767 (1.701, 1.835) | <0.001 |
| High genetic risk | 12,670/74,055 | 2.917 (2.817, 3.021) | <0.001 | 2.929 (2.828, 3.033) | <0.001 | 3.08 (2.972, 3.193) | <0.001 |

Note: Data was presented as odds ratio (95% confidence interval). Model 1 was adjusted for sex and age. Model 2 was adjusted for the terms in model 1, household income, education qualification, smoking status, drinking frequency, and diet quality. Model 3 was adjusted for terms in model 2, hypertension, diabetes, obesity, and high cholesterol.

# Table S7. The stratified associations of lifetime shift work frequency with gout risk in the subgroups.

| **Subgroup** | **None** | **< 3/month** | **3-8/month** | **> 8/month** | **P for interaction** |
| --- | --- | --- | --- | --- | --- |
| **Sex** |  |  |  |  | 0.839 |
| Men | 1 | 1.243 (0.89, 1.737) | 1.025 (0.816, 1.288) | 1.152 (0.957, 1.387) |  |
| Women | 1 | 0.945 (0.3, 2.975) | 0.823 (0.43, 1.573) | 0.829 (0.466, 1.474) |  |
| **Age** |  |  |  |  | 0.449 |
| <60 | 1 | 1.29 (0.879, 1.895) | 0.918 (0.703, 1.199) | 1.025 (0.822, 1.277) |  |
| ≥60 | 1 | 0.989 (0.55, 1.779) | 1.144 (0.795, 1.646) | 1.248 (0.933, 1.669) |  |
| **Smoking status** |  |  |  |  | 0.440 |
| Never | 1 | 1.404 (0.892, 2.208) | 0.795 (0.562, 1.124) | 1.135 (0.872, 1.479) |  |
| Past | 1 | 1.164 (0.721, 1.88) | 1.174 (0.865, 1.592) | 1.128 (0.873, 1.456) |  |
| Current | 1 | 0.554 (0.134, 2.292) | 1.219 (0.633, 2.349) | 0.918 (0.5, 1.683) |  |
| **Drinking frequency** |  |  |  |  | 0.253 |
| Never | 1 | 0.001 (0, 3.28364390930084E+31) | 1.155 (0.331, 4.026) | 1.265 (0.447, 3.575) |  |
| Infrequent | 1 | 0.732 (0.23, 2.331) | 1.362 (0.8, 2.319) | 0.83 (0.484, 1.424) |  |
| Frequent | 1 | 1.293 (0.925, 1.806) | 0.929 (0.731, 1.181) | 1.139 (0.943, 1.376) |  |
| **Diet score** |  |  |  |  | 0.458 |
| Poor | 1 | 0.795 (0.189, 3.349) | 0.539 (0.164, 1.774) | 0.99 (0.464, 2.114) |  |
| Intermediate | 1 | 0.946 (0.463, 1.935) | 0.834 (0.522, 1.333) | 1.341 (0.966, 1.863) |  |
| Healthy | 1 | 1.323 (0.913, 1.917) | 1.077 (0.839, 1.381) | 1.01 (0.811, 1.257) |  |
| **Sleep duration** |  |  |  |  | 0.233 |
| Short | 1 | 0.742 (0.327, 1.683) | 1.136 (0.756, 1.707) | 0.927 (0.65, 1.321) |  |
| Normal | 1 | 1.285 (0.892, 1.852) | 0.941 (0.723, 1.224) | 1.187 (0.964, 1.461) |  |
| Long | 1 | 2.679 (0.79, 9.084) | 1.024 (0.386, 2.718) | 1.068 (0.455, 2.508) |  |
| **Chronotype** |  |  |  |  | 0.097 |
| Early | 1 | 1.603 (1.074, 2.394) | 1.132 (0.852, 1.503) | 1.097 (0.856, 1.407) |  |
| Late | 1 | 0.638 (0.315, 1.293) | 0.749 (0.498, 1.126) | 1.096 (0.823, 1.46) |  |
| **Education qualification** |  |  |  |  | 0.434 |
| College or above | 1 | 1.321 (0.81, 2.153) | 0.947 (0.658, 1.363) | 1.198 (0.89, 1.613) |  |
| High school | 1 | 1.2 (0.776, 1.855) | 0.904 (0.674, 1.213) | 1.078 (0.857, 1.356) |  |
| Less than high school | 1 | 0.419 (0.057, 3.088) | 1.841 (0.911, 3.719) | 0.929 (0.467, 1.851) |  |
| **Household income** |  |  |  |  | 0.670 |
| Low | 1 | 0.389 (0.048, 3.12) | 0.726 (0.218, 2.422) | 1.12 (0.535, 2.343) |  |
| Medium | 1 | 0.849 (0.504, 1.43) | 1.004 (0.743, 1.356) | 1.044 (0.816, 1.336) |  |
| High | 1 | 1.771 (1.148, 2.733) | 0.965 (0.684, 1.361) | 1.174 (0.884, 1.559) |  |
| **Diabetes status** |  |  |  |  | 0.667 |
| No | 1 | 1.16 (0.828, 1.626) | 0.979 (0.784, 1.222) | 1.128 (0.943, 1.35) |  |
| Yes | 1 | 1.828 (0.63, 5.304) | 1.327 (0.537, 3.284) | 0.627 (0.253, 1.55) |  |
| **Hypertension** |  |  |  |  | 0.963 |
| No | 1 | 1.126 (0.679, 1.868) | 0.931 (0.67, 1.294) | 1.032 (0.787, 1.352) |  |
| Yes | 1 | 1.229 (0.812, 1.862) | 1.03 (0.775, 1.369) | 1.141 (0.906, 1.438) |  |
| **Obese** |  |  |  |  | 0.718 |
| No | 1 | 1.044 (0.673, 1.619) | 0.981 (0.739, 1.302) | 1.136 (0.901, 1.433) |  |
| Yes | 1 | 1.439 (0.897, 2.307) | 1.014 (0.728, 1.414) | 1.06 (0.811, 1.387) |  |
| **High cholesterol** |  |  |  |  | 0.439 |
| No | 1 | 1.135 (0.659, 1.955) | 0.817 (0.546, 1.221) | 0.981 (0.721, 1.335) |  |
| Yes | 1 | 1.235 (0.83, 1.838) | 1.082 (0.838, 1.397) | 1.165 (0.941, 1.443) |  |

Note: Data was adjusted for sex, age, smoking status, drinking frequency, diet quality score, sleep duration, chronotype, education qualification, household income, diabetes, hypertension, obesity, and high cholesterol. CI, confidence interval; HR, hazard ratio.

# Table S8. The stratified associations of lifetime shift work duration with gout risk in the subgroups.

| **Subgroup** | **None** | **< 5 years** | **5-10 years** | **> 10 years** | **P for interaction** |
| --- | --- | --- | --- | --- | --- |
| **Sex** |  |  |  |  | 0.788 |
| Men | 1 | 1.22 (0.989, 1.505) | 1 (0.741, 1.351) | 1.087 (0.888, 1.33) |  |
| Women | 1 | 0.803 (0.407, 1.585) | 0.805 (0.354, 1.832) | 0.891 (0.479, 1.658) |  |
| **Age** |  |  |  |  | 0.283 |
| <60 | 1 | 1.207 (0.946, 1.541) | 0.84 (0.586, 1.205) | 0.951 (0.748, 1.207) |  |
| ≥60 | 1 | 1.075 (0.757, 1.527) | 1.196 (0.761, 1.88) | 1.269 (0.922, 1.745) |  |
| **Smoking status** |  |  |  |  | 0.574 |
| Never | 1 | 1.231 (0.92, 1.647) | 0.852 (0.541, 1.34) | 0.961 (0.713, 1.295) |  |
| Past | 1 | 1.234 (0.923, 1.65) | 1.125 (0.761, 1.664) | 1.087 (0.822, 1.438) |  |
| Current | 1 | 0.565 (0.225, 1.416) | 0.794 (0.314, 2.009) | 1.384 (0.781, 2.453) |  |
| **Drinking frequency** |  |  |  |  | 0.805 |
| Never | 1 | 1.265 (0.366, 4.376) | 1.349 (0.305, 5.969) | 0.807 (0.226, 2.882) |  |
| Infrequent | 1 | 1.316 (0.763, 2.27) | 0.896 (0.389, 2.064) | 0.824 (0.467, 1.454) |  |
| Frequent | 1 | 1.139 (0.916, 1.418) | 0.962 (0.709, 1.306) | 1.091 (0.888, 1.341) |  |
| **Diet score** |  |  |  |  | 0.392 |
| Poor | 1 | 1.379 (0.63, 3.018) | 0.286 (0.039, 2.112) | 0.626 (0.24, 1.629) |  |
| Intermediate | 1 | 1.142 (0.762, 1.713) | 1.236 (0.735, 2.077) | 1.046 (0.715, 1.53) |  |
| Healthy | 1 | 1.161 (0.912, 1.478) | 0.93 (0.661, 1.308) | 1.063 (0.844, 1.339) |  |
| **Sleep duration** |  |  |  |  | 0.026 |
| Short | 1 | 0.836 (0.528, 1.323) | 1.231 (0.751, 2.017) | 0.972 (0.668, 1.413) |  |
| Normal | 1 | 1.298 (1.032, 1.632) | 0.915 (0.648, 1.292) | 1.042 (0.824, 1.316) |  |
| Long | 1 | 1.074 (0.405, 2.848) | 0.002 (0, 1525242635.362) ### | 1.866 (0.869, 4.01) |  |
| **Chronotype** |  |  |  |  | 0.248 |
| Early | 1 | 1.281 (0.977, 1.68) | 1.029 (0.703, 1.507) | 1.142 (0.879, 1.484) |  |
| Late | 1 | 1.084 (0.771, 1.523) | 0.749 (0.443, 1.267) | 0.868 (0.62, 1.216) |  |
| **Education qualification** |  |  |  |  | 0.837 |
| College or above | 1 | 1.225 (0.909, 1.652) | 0.75 (0.439, 1.282) | 1.208 (0.854, 1.708) |  |
| High school | 1 | 1.111 (0.837, 1.475) | 1.056 (0.742, 1.501) | 0.971 (0.759, 1.243) |  |
| Less than high school | 1 | 0.991 (0.381, 2.583) | 1.229 (0.427, 3.532) | 1.166 (0.614, 2.214) |  |
| **Household income** |  |  |  |  | 0.804 |
| Low | 1 | 1.037 (0.415, 2.592) | 1.394 (0.475, 4.089) | 0.625 (0.232, 1.688) |  |
| Medium | 1 | 1.122 (0.835, 1.507) | 0.79 (0.516, 1.211) | 1.019 (0.786, 1.321) |  |
| High | 1 | 1.256 (0.932, 1.691) | 1.153 (0.759, 1.753) | 1.086 (0.784, 1.504) |  |
| **Diabetes status** |  |  |  |  | 0.547 |
| No | 1 | 1.176 (0.957, 1.444) | 0.974 (0.729, 1.301) | 1.047 (0.86, 1.274) |  |
| Yes | 1 | 0.944 (0.361, 2.473) | 0.866 (0.259, 2.89) | 1.092 (0.475, 2.511) |  |
| **Hypertension** |  |  |  |  | 0.807 |
| No | 1 | 1.171 (0.868, 1.58) | 0.817 (0.524, 1.276) | 0.96 (0.711, 1.295) |  |
| Yes | 1 | 1.152 (0.88, 1.507) | 1.069 (0.742, 1.538) | 1.102 (0.86, 1.414) |  |
| **Obese** |  |  |  |  | 0.956 |
| No | 1 | 1.141 (0.88, 1.48) | 1.019 (0.71, 1.462) | 1.023 (0.787, 1.329) |  |
| Yes | 1 | 1.203 (0.879, 1.647) | 0.89 (0.568, 1.396) | 1.082 (0.816, 1.434) |  |
| **High cholesterol** |  |  |  |  | 0.081 |
| No | 1 | 0.831 (0.56, 1.233) | 0.758 (0.448, 1.282) | 1.12 (0.817, 1.537) |  |
| Yes | 1 | 1.345 (1.065, 1.698) | 1.076 (0.771, 1.502) | 1.008 (0.793, 1.283) |  |

Note: Data was adjusted for sex, age, smoking status, drinking frequency, diet quality score, sleep duration, chronotype, education qualification, household income, diabetes, hypertension, obesity, and high cholesterol. ### The wide confidence interval suggests substantial uncertainty in the estimate, likely due to the absence of outcome events in this group. CI, confidence interval; HR, hazard ratio.

# Table S9. Adjusted HRs (95% CIs) for current and lifetime shift work and incident gout, further adjusted for chronotype (Model 4), sleep duration (Model 5), and chronic kidney disease and diuretic use (Model 6).

| **Shift work schedule** | **Incident cases/N** | **Model 4** | | **Model 5** | | **Model 6** | |
| --- | --- | --- | --- | --- | --- | --- | --- |
|  |  | **HR (95% CI)** | **P** | **HR (95% CI)** | **P** | **HR (95% CI)** | **P** |
| **Current shift work** |  |  |  |  |  |  |  |
| Day worker | 3,422/232,948 | Ref | - | Ref | - | Ref | - |
| Shift but never/rarely night shifts | 391/23,838 | 1.063 (0.957, 1.182) | 0.256 | 1.062 (0.955, 1.181) | 0.266 | 1.054 (0.948, 1.172) | 0.331 |
| Some night shifts | 284/13,876 | 1.15 (1.017, 1.3) | 0.026 | 1.15 (1.017, 1.3) | 0.026 | 1.152 (1.019, 1.303) | 0.024 |
| Usual/permanent night shifts | 185/10,838 | 0.945 (0.813, 1.098) | 0.458 | 0.949 (0.817, 1.103) | 0.499 | 0.941 (0.810, 1.093) | 0.424 |
| **Lifetime night shift work** |  |  | |  | |  | |
| **Average lifetime night shift frequency** |  |  |  |  |  |  |  |
| None | 658/56,884 | Ref | - | Ref | - | Ref | - |
| <3/month | 40/2,067 | 1.197 (0.869, 1.649) | 0.272 | 1.2 (0.871, 1.653) | 0.265 | 1.195 (0.867, 1.647) | 0.276 |
| 3-8/month | 97/6,845 | 0.993 (0.801, 1.231) | 0.949 | 0.989 (0.797, 1.226) | 0.918 | 0.982 (0.792, 1.218) | 0.869 |
| >8/month | 165/9,117 | 1.096 (0.919, 1.306) | 0.308 | 1.102 (0.924, 1.313) | 0.28 | 1.094 (0.918, 1.304) | 0.313 |
| **Lifetime duration of night shift work** |  |  |  |  |  |  |  |
| None | 658/56,884 | Ref | - | Ref | - | Ref | - |
| <5 years | 114/6,432 | 1.164 (0.953, 1.422) | 0.136 | 1.163 (0.952, 1.421) | 0.139 | 1.147 (0.939, 1.401) | 0.18 |
| 5-10 years | 53/3,979 | 0.963 (0.727, 1.276) | 0.793 | 0.962 (0.726, 1.275) | 0.787 | 0.971 (0.733, 1.287) | 0.836 |
| >10 years | 135/7,618 | 1.045 (0.863, 1.265) | 0.655 | 1.05 (0.867, 1.272) | 0.615 | 1.043 (0.862, 1.263) | 0.663 |

Note: Data was presented as hazard ratios (95% confidence interval). Model 3 was adjusted for sex, age, household income, education qualification, smoking status, drinking frequency, diet quality, hypertension, diabetes, obesity, and high cholesterol. Model 4 was adjusted for the covariates in Model 3 plus chronotype. Model 5 was adjusted for the covariates in Model 3 plus sleep duration. Model 6 was adjusted for the covariates in Model 3 plus chronic kidney disease and diuretic use. CI, confidence interval; HR, hazard ratio.

# Table S10. Adjusted HRs (95% CIs) for current and lifetime shift work and incident gout, based on hospital inpatient data (ICD-9 and ICD-10 codes).

| **Shift work schedule** | **Incident cases/N** | **Gout cases based on hospital inpatient data (ICD-9 and ICD-10 codes)** | |
| --- | --- | --- | --- |
|  |  | **HR (95% CI)** | **P** |
| **Current work schedule** |  |  |  |
| Day worker | 1,441/230,967 | Ref | - |
| Shift but never/rarely night shifts | 178/23,625 | 1.126 (0.962, 1.318) | 0.14 |
| Some night shifts | 129/13,721 | 1.259 (1.048, 1.511) | 0.014 |
| Usual/permanent night shifts | 74/10,727 | 0.9 (0.710, 1.140) | 0.384 |
| **Lifetime night shift work** |  |  | |
| **Average lifetime night shift frequency** |  |  |  |
| None | 247/56,473 | Ref | - |
| <3/month | 19/2,046 | 1.5 (0.94, 2.4) | 0.089 |
| 3-8/month | 37/6,785 | 1.03 (0.727, 1.46) | 0.864 |
| >8/month | 66/9,018 | 1.16 (0.88, 1.54) | 0.287 |
| **Lifetime duration of night shift work** |  |  |  |
| None | 247/56,473 | Ref | - |
| <5 years | 47/6,365 | 1.27 (0.926, 1.74) | 0.139 |
| 5-10 years | 27/3,953 | 1.32 (0.881, 1.96) | 0.18 |
| >10 years | 48/7,531 | 0.999 (0.726, 1.37) | 0.994 |

Note: Data was presented as hazard ratios (95% confidence interval). Model was adjusted for sex, age, household income, education qualification, smoking status, drinking frequency, diet quality, hypertension, diabetes, obesity, and high cholesterol. CI, confidence interval; HR, hazard ratio; ICD-9, International Classification of Diseases, 9th Revision; ICD-10, International Classification of Diseases, 10th Revision.

# Table S11. Adjusted HRs (95% CIs) for current and lifetime shift work and incident gout, excluding self-reported cases if SUA < 360 μmol/L and did not report receiving ULT.

| **Shift work schedule** | **Incident cases/N** | **Gout cases excluding self-reported cases if SUA < 360 μmol/L and did not report receiving ULT** | |
| --- | --- | --- | --- |
|  |  | **HR (95% CI)** | **P** |
| **Current work schedule** |  |  |  |
| Day worker | 3,098/232,624 | Ref | - |
| Shift but never/rarely night shifts | 345/23,792 | 1.04 (0.931, 1.17) | 0.475 |
| Some night shifts | 272/13,864 | 1.2 (1.06, 1.37) | 0.004 |
| Usual/permanent night shifts | 172/10,825 | 0.97 (0.83, 1.13) | 0.702 |
| **Lifetime night shift work** |  |  | |
| **Average lifetime night shift frequency** |  |  |  |
| None | 579/56,805 | Ref | - |
| <3/month | 37/2,064 | 1.24 (0.887, 1.73) | 0.210 |
| 3-8/month | 89/6,837 | 1.03 (0.822, 1.29) | 0.798 |
| >8/month | 156/9,108 | 1.17 (0.972, 1.4) | 0.099 |
| **Lifetime duration of night shift work** |  |  |  |
| None | 579/56,805 | Ref | - |
| <5 years | 108/6,426 | 1.23 (1, 1.52) | 0.047 |
| 5-10 years | 48/3,974 | 0.987 (0.734, 1.33) | 0.932 |
| >10 years | 126/7,609 | 1.1 (0.902, 1.34) | 0.345 |

Note: Data was presented as hazard ratios (95% confidence interval). Model was adjusted for sex, age, household income, education qualification, smoking status, drinking frequency, diet quality, hypertension, diabetes, obesity, and high cholesterol. CI, confidence interval; HR, hazard ratio; SUA, serum uric acid; ULT: urate-lowering therapy.

# Table S12. Adjusted ORs (95% CIs) for current and lifetime shift work and prevalent hyperuricemia, further adjusted for chronotype (Model 4), sleep duration (Model 5), and chronic kidney disease and diuretic use (Model 6).

| **Shift work schedule** | **Incident cases/N** | **Model 4** | | **Model 5** | | **Model 6** | |
| --- | --- | --- | --- | --- | --- | --- | --- |
|  |  | **OR (95% CI)** | **P** | **OR (95% CI)** | **P** | **OR (95% CI)** | **P** |
| **Current shift work** |  |  |  |  |  |  |  |
| Day worker | 25,086/221,620 | Ref | - | Ref | - | Ref | - |
| Shift but never/rarely night shifts | 2,715/22,681 | 0.994 (0.951, 1.039) | 0.791 | 0.993 (0.949, 1.038) | 0.74 | 0.992 (0.949, 1.04) | 0.731 |
| Some night shifts | 1,944/13,289 | 1.108 (1.051, 1.169) | < 0.001 | 1.106 (1.049, 1.167) | < 0.001 | 1.11 (1.05, 1.17) | < 0.001 |
| Usual/permanent night shifts | 1,521/10,302 | 1.106 (1.042, 1.174) | 0.001 | 1.11 (1.046, 1.178) | 0.001 | 1.11 (1.05, 1.18) | < 0.001 |
| **Lifetime night shift work** |  |  | |  | |  |  |
| **Average lifetime night shift frequency** |  |  |  |  |  |  |  |
| None | 5,226/54,199 | Ref | - | Ref | - | Ref |  |
| <3/month | 254/1,978 | 1.083 (0.94, 1.249) | 0.269 | 1.084 (0.94, 1.249) | 0.268 | 1.09 (0.941, 1.25) | 0.248 |
| 3-8/month | 767/6,570 | 1.029 (0.945, 1.12) | 0.515 | 1.027 (0.943, 1.118) | 0.543 | 1.02 (0.938, 1.11) | 0.616 |
| >8/month | 1,141/8,762 | 1.034 (0.96, 1.113) | 0.375 | 1.032 (0.959, 1.111) | 0.398 | 1.04 (0.964, 1.12) | 0.316 |
| **Lifetime duration of night shift work** |  |  |  |  |  |  |  |
| None | 5,226/54,199 | Ref | - | Ref | - | Ref |  |
| <5 years | 773/6,142 | 1.094 (1.004, 1.191) | 0.039 | 1.091 (1.002, 1.188) | 0.045 | 1.09 (0.999, 1.19) | 0.051 |
| 5-10 years | 386/3,831 | 0.885 (0.789, 0.992) | 0.037 | 0.884 (0.788, 0.992) | 0.036 | 0.889 (0.791, 0.995) | 0.044 |
| >10 years | 1,003/7,337 | 1.068 (0.987, 1.155) | 0.101 | 1.067 (0.986, 1.154) | 0.106 | 1.07 (0.99, 1.16) | 0.087 |

Note: Data was presented as odds ratios (95% confidence interval). Model 3 was adjusted for sex, age, household income, education qualification, smoking status, drinking frequency, diet quality, hypertension, diabetes, obesity, and high cholesterol. Model 4 was adjusted for the covariates in Model 3 plus chronotype. Model 5 was adjusted for the covariates in Model 3 plus sleep duration. Model 6 was adjusted for the covariates in Model 3 plus chronic kidney disease and diuretic use. CI, confidence interval; HR, hazard ratio.
